# Dynamic workforce modulation and foraging efficiency in Eusocial insect colonies

**DOI:** 10.1371/journal.pcbi.1014258

**Published:** 2026-10-09

**Authors:** Daniel Campos, Javier Cristín, Pol Fernández-López, Roger Lloret-Cabot, Meritxell Genovart, Frederic Bartumeus

**Affiliations:** 1 Grup de Física Estadística, Departament de Física. Facultat de Ciències, Universitat Autònoma de Barcelona, Bellaterra (Barcelona), Spain; 2 Centre d’Estudis Avançats de Blanes (CEAB-CSIC), Blanes, Girona, Spain; 3 ICREA, Institut Català de Recerca i Estudis Avançats, Barcelona, Spain; 4 Centre de Recerca Ecològica i Aplicacions Forestals (CREAF), Cerdanyola del Vallès, Barcelona, Spain; Kiel University, GERMANY

## Abstract

Understanding the fitness advantages conferred by eusociality remains a central challenge in behavioral ecology. One promising approach is to identify collective strategies that shift efficiency within social groups. Here, we explore the hypothesis that reserve workforces in ant colonies represent an adaptive mechanism that enhances flexibility and foraging efficiency under fluctuating environmental conditions. We examine how departure and return rates of foragers modulate such reserve workers and how these time-dependent dynamics shape the colony’s overall energetic balance. By integrating an energetic-balance framework with stochastic search simulations inspired by empirical results from four colonies of *Aphaenogaster senilis* ants, we quantify the energetic requirements for colony viability, incorporating energy intake, search costs, and basal metabolic demands. Our results show that as colonies grow, maintaining a positive energy balance requires a disproportionately larger relative workforce. By modulating departure and return rates over time, colonies control the synchrony of their collective search and efficiently activate or suppress their reserve workforce to scale foraging effort as needed. These findings suggest that the “lazy” or weakly engaged workers commonly observed in large colonies function as an essential reserve that stabilizes colony energetics and enhances responsiveness. Together, our results provide a functional explanation for sublinear metabolic scaling in eusocial groups and highlight workforce modulation as a key factor underlying their energetic stability and evolutionary success.

## Author summary

Understanding how social insect colonies balance energy expenditure with resource acquisition is central to explaining their ecological success. Here, we develop a theoretical framework that links (i) energetic constraints necessary to reach a positive energy balance in the colony, to (ii) the proportion of workers actively engaged in foraging. Our results analyze the fundamental trade-off between increasing the number of active foragers to improve resource discovery, and the corresponding increase in the metabolic costs. As colonies grow, the latter becomes increasingly important, so strategies for regulating the fraction of workers actively engaged in foraging becomes critical. As a solution to this, large colonies would benefit from maintaining a reserve of inactive workers that can be recruited whenever necessary. This provides a functional explanation for the widespread presence of apparently “lazy” individuals in eusocial societies, suggesting that they contribute to colony resilience rather than representing inefficiency. The model predicts how environmental conditions influence colony size and search strategies, and offers testable hypotheses linking task allocation, metabolic scaling, and collective behaviour. More broadly, our findings highlight workforce redundancy as a mechanism supporting the energetic stability and evolutionary success of complex social systems.

## 1. Introduction

Eusociality provides biological species with a fundamentally expanded repertoire of adaptive strategies, offering substantial advantages in ecological and evolutionary contexts. This view is reinforced by genetic evidence showing that such a complex form of social organization has arisen independently and repeatedly throughout evolutionary history (see [1–6] for recent accounts). Additionally, phylogenetic methods provide evidence that the transition from solitary to collective strategies, resulting from colony growth/development is rarely reversed [4]. As a central component of this expanded adaptive repertoire, dynamic task allocation and colony-level activation have been experimentally shown to rely on regulatory mechanisms that respond to external fluctuations or disturbances, thereby providing colonies with substantial behavioral flexibility [7]. In line with this, ant colonies can shift from individual to collective foraging strategies depending on resource size [8], density [9–12], and quality [13]. Moreover, increases in group size typically favor collective over individual foraging strategies [13,14].

However, eusociality also entails that the intrinsic value of any given individual decreases as group size increases. Intriguingly, this promotes a form of redundancy within colonies, whereby active workers coexist with inactive individuals that remain available for rapid recruitment when specific tasks arise [15]. The proportion of these “inactive” or “lazy” workers has been shown to increase with colony size [16–21]. Although maintaining such inactive individuals entails non-trivial metabolic costs, several hypotheses have been proposed to explain their persistence [20]. The most strongly supported view interprets these workers as a reserve workforce that equips the colony with the capacity for rapid adaptive responses to transient demands or extreme events [22,23].

More broadly, the use of redundant search units—formalized as the redundancy principle [24]—has been proposed as an evolutionary adaptation across a wide range of biological and chemical systems [25–31]. The central idea is straightforward: increasing the number of searchers, whether molecules or organisms, reduces the expected search time for targets, and natural selection should therefore favor redundancy whenever feasible. However, in the context of social foraging, energetic constraints become critical, as deploying an arbitrarily large workforce may be metabolically inefficient. This trade-off suggests that colonies must regulate how many workers are active at any given time, making the dynamic modulation of task-related workforces a potentially essential component of the transition from individual to collective behavior. It also highlights the importance of understanding how colonies balance the benefits of redundancy with the energetic costs of maintaining and activating additional workers. Although empirical evidence is still limited, several studies indicate that such modulation enables colonies to maintain—or rapidly restore—overall activity levels even after the removal of a substantial fraction of active workers. This compensatory response has been documented in *Temnothorax rugatulus* [16], Myrmica kotokui [32], and in bees [33]. Quantifying the specific benefits that workforce modulation and the emergence of redundancy provide to ant colonies therefore represents a key step toward understanding the functional basis of eusociality.

In the present work, we (i) provide numerical evidence (through simulations reproducing colonies foraging patterns) that dynamic workflow allocation acts as a convenient—and potentially necessary—mechanism conferring additional behavioral flexibility to eusocial groups, (ii) examine the conditions under which such enhanced flexibility compensates for the metabolic costs associated with maintaining inactive workers, and (iii) contrast such findings against empirical datasets corresponding to foraging experiments of ant colonies under different arenas/conditions.

More specifically, we investigate how the time-dependent modulation of active foragers affect search efficiency, quantified through the energetic balance resulting from foraging activity. Mathematically, previous work has shown that for a group of *N* diffusive individuals starting their search at *t* = 0 from a common place (e.g., the nest), the time required to reach a single target located at distance $d_{r}$ from the nest scales as $\langle T\rangle \sim d_{r}^{2}/\ln N$, provided $d_{r}$ and *N* are large enough (see [34–39] for a comprehensive survey, and [40–42] for the latest advances). Here, we extend this classical framework by considering a more realistic scenario in which the number of active searchers varies in time. We denote by $N_{a}(t)$ the number of active workers, which become active or inactive at rates $\gamma_{b}$ and $\gamma_{d}$, respectively (with subscripts *b* and *d* referring to “birth” and “death” of search trajectories). In this formulation, the limit $\gamma_{b}\to 0$ corresponds to highly ‘asynchronous’ search (this is, a search pattern where individuals leave the nest sporadically and so we rarely have several searchers active simultaneously). The contrary limit $\gamma_{b}\to \infty$ represents then fully ‘synchronous’ collective search where many searchers stay active simultaneously. Once a searcher completes a trajectory (at rate $\gamma_{d}$) it becomes inactive again (effectively returning to the nest) so individuals continuously switch between active and inactive states until the search concludes. In previous work [43], we identified the optimal values of $\gamma_{b}$ (for $\gamma_{d}\to 0$) that minimize the collective search cost required to reach the target. In the present study, we substantially generalize this idea by incorporating energetic gains and costs relevant to eusocial colonies. Our goal is to determine under which conditions the presence of reserve workforces becomes particularly advantageous, and how the interplay between activation rates, deactivation rates, and energetic constraints shapes the efficiency of collective search.

## 2. Results

In the following we (i) analyze empirical datasets to characterize typical patterns of birth and death rates exhibited by real colonies (in Section 2.1), and then (ii) we take advantage of such empirical patterns to run the foraging model above and extract conclusions about the corresponding energy balance of colonies as a function of the size of the colony and/or the workload levels used (in Sections 2.2 and 2.3).

### 2.1. Time evolution of active foragers in the arena

We quantified the time evolution of departures (birth rate, $\gamma_{b}$) and returns (death rate, $\gamma_{d}$) of foraging ants in the two experimental arenas (Fig 1). Colonies exhibited a gradual increase in the number of ants entering the arena, a pattern driven by rising birth rate—more or less pronounced depending on the colony and the experiment—and by consistently decreasing death rates. The latter aligns with a strategy commonly reported in many ant species: exploration begins with one or a few scouts that assess external conditions and help the colony evaluate potential risks outside the nest. Only once safety is established does the number of searching individuals, and the diversity of their trajectories, expand. Highly nonstationary patterns are thus the rule rather than the exception in ant foraging dynamics, rendering direct comparison with mathematical models non-trivial. This precludes, for instance, a straightforward quantitative comparison with our previous results [43], in which activation and deactivation rates were held constant. The present work instead aims to establish whether, and through what mechanisms, the observed temporal trends in these rates shape the colony’s overall energetic balance.

**Fig 1:**
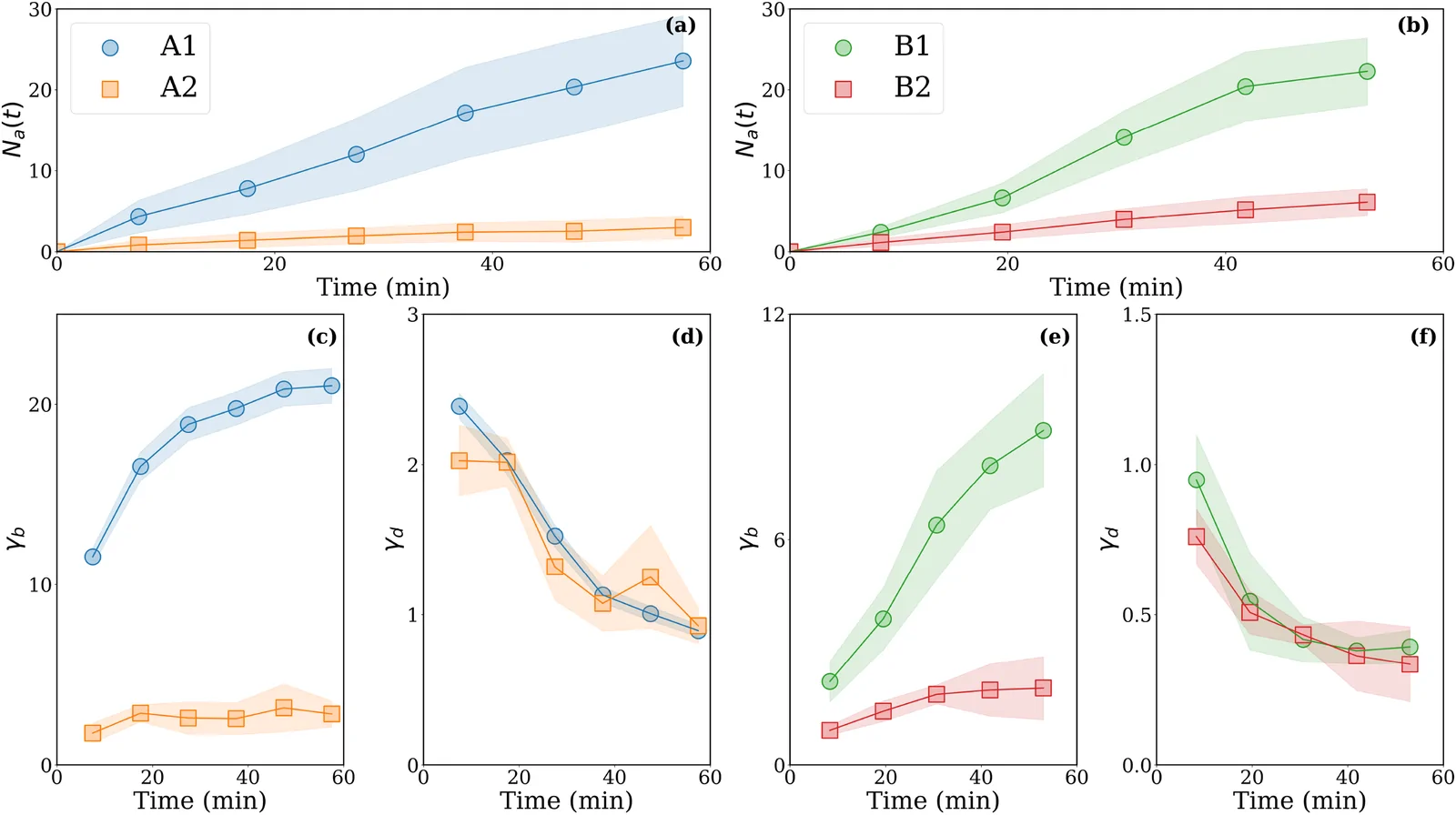
Average number of ants $N_{a}(t)$ and rates $\gamma_{b}$ and $\gamma_{d}$ obtained from the experimental datasets for the four colonies considered, two for experiment A (left) and two for experiment B (right). Only data from the first 60 minutes of the foraging process is considered, which corresponds in most cases to the search phase and the initial part of the food recollection phase. Error bars (shaded region) correspond to the 95% confidence interval of the mean, computed from all daily experiments carried out for each colony.

Experiment A (Fig 1a,1c,1d) allows us to compare the foraging strategies (identified through the dynamics of rates $\gamma_{d}$ and $\gamma_{b}$) used by two relatively small colonies of different size. It is seen that there is a clear tendency of $\gamma_{b}$ ($\gamma_{d}$) to increase (decrease) throughout the foraging process. Furthermore, the larger colony (A1) also exhibits a higher activity in terms of the number of active workers (note that $N_{a}$ for colony A1 is about six times that for colony A2, though its size $N_{col}$ is only ≈2.5 times larger). Finally, it is also evident that the larger activity of colony A1 is orchestrated through a much larger number of search trajectories (evidenced through larger birth rates $\gamma_{b}$), while durations of these trajectories are approximately the same for both colonies (this is, their $\gamma_{d}$ rates do not vary significantly).

Colonies B1 and B2 in experiment B (Fig 1b,1e,1f), have approximately the same size. However, we observed a large variability between their foraging activities throughout the experiments (the number of active foragers $N_{a}$ was on average two times larger for B1). This may be due to the history of the two colonies (they may be originally adapted to different conditions before the experiments, or their task partitioning/needs may be different). Whatever the specific reason, different foraging activities $N_{a}(t)$ may presumably reflect different workforces $N_{w}$, too.

In experiments B, we found similar results for the rates despite using a different arena and food conditions. We identified a clear tendency to increase $\gamma_{b}$ values and decrease $\gamma_{d}$ values with time during the foraging process; this seems to be a rather robust dynamics through all colonies and conditions considered. Besides, the difference in foraging activities between colonies B1 and B2 results again from differences in $\gamma_{b}$ (rather than $\gamma_{d}$, which shows similar values for both colonies).

According to theoretical results in [43] (plus additional numerical tests we have carried out) dimensionality does not affect qualitatively the results on the search efficiency of groups, but only quantitatively. One would then expect that results from Experiments A (in one dimensional arenas) and B (two-dimensional) are then comparable and show similar patterns, although with different $\gamma_{b}$ and $\gamma_{d}$ values due to the different conditions.

We note that although Fig 1 only shows averaged rates, the intraday (morning *vs.* afternoon) or interday variability observed between experiments does not significantly affect our conclusions above (in Supplementary Information File S1 Text we provide and discuss the corresponding results for the sake of completeness). Extending our results to later phases of foraging (gathering and pos-gathering stages) shows that once the entire arena has been explored and no additional food resources are available, the colony tends to progressively decrease the number of active workers in the arena, reflected by a slight increase in $\gamma_{d}$. These trends, which are also presented in the Supplementary Information File S1 Text, are mostly evident in colonies B1 and B2, while colonies A1 and A2 did not reach such a decline in activity during the duration of the experiments.

Finally, we stress that food conditioning does not seem to induce any significant trend or variation in the strategies of the different colonies, at least in terms of $\gamma_{b}$ and $\gamma_{d}$. This is true even in the case of colonies B1 and B2, where food amount and disposition in the arena was kept the same for two/three consecutive weeks of experiments [44]. So, we find no evidence of adaptation to resource distributions throughout the experiments. This may be because the differences in food spatial allocation used in our experiments were not sufficiently large. An alternative explanation would be that the adaptation of collective strategies to resource distributions, if any, requires longer (maybe pluriannual) timescales. Specific experiments should then be necessary to confirm or discard these ideas.

### 2.2. Energy balance optimization of collective searches through $\gamma_{b}$ and $\gamma_{d}$

In Fig 2, we show heatmaps of the average energy balance $\Delta E^{*}$. The quantity $\Delta E^{*}$ is computed through random-walk search simulations that allow us to determine $T_{fp}$ and $T_{C}$, as a function of the departure and return rates $\gamma_{b}$ and $\gamma_{d}$, and for a specific choice of the parameters $\alpha_{i}$ and $\alpha_{f}$ (see S1 Text for explicit plots of these magnitudes). The values of the parameters were selected to ensure that we explore the space parameter around $\Delta E^{*}\approx 0$, so that we have a clear transition between a positive and a negative energy balance. Alternative choices of $\alpha_{i}$ and $\alpha_{f}$ were also explored, and we verified that they do not qualitatively alter our results. All simulation results were averaged over 10^3^ realizations, using a time step Δt=0.01 in the simulation scheme described in Sec. 4.2.

**Fig 2:**
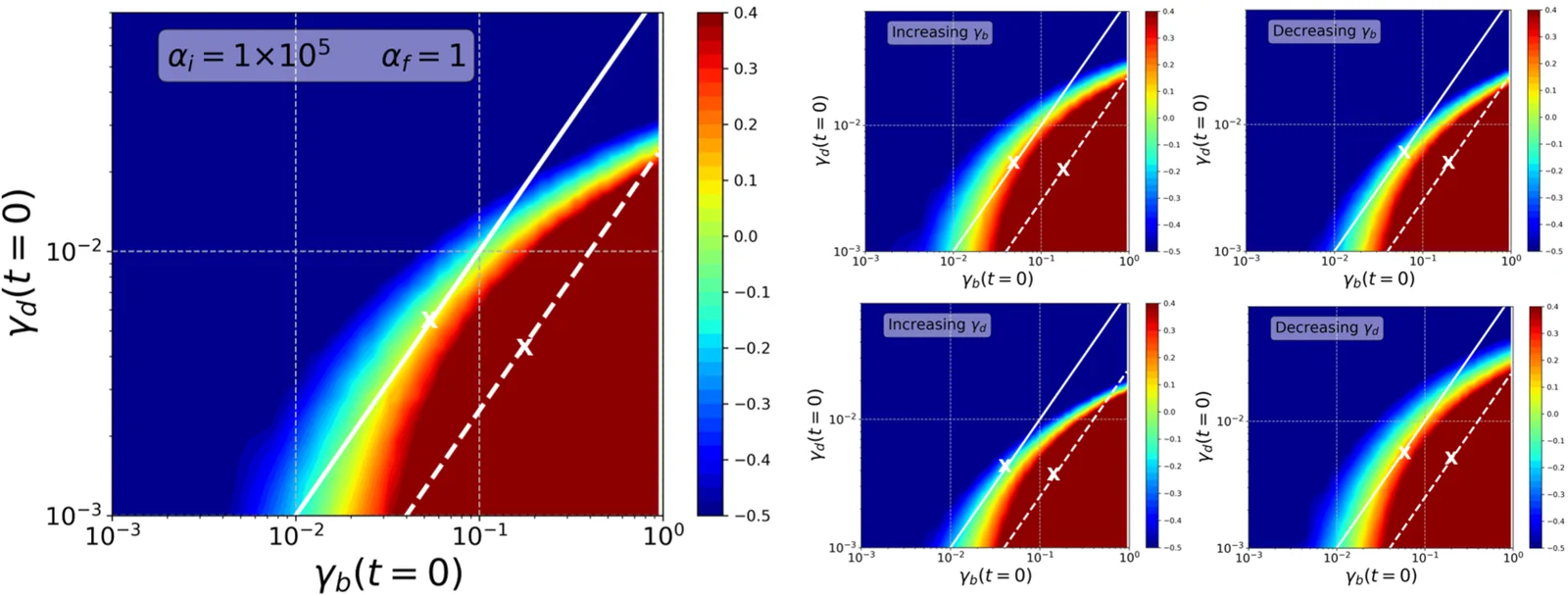
Heatmaps obtained from random-walk simulations for the energy balance $\Delta E^{*}$ as a function of rates $\gamma_{b}$, $\gamma_{d}$ (using $d_{r}=10$, *v* = 1, $d_{d}=1$). (Left): reference case for specific values of $\alpha_{i}$, $\alpha_{f}$ (indicated in the legend), chosen so the shape of the region $\Delta E^{*}>0$ where the colony would be energetically viable can be identified. (Right): case when $\gamma_{b}(t)$ or $\gamma_{d}(t)$ are allowed to increase or decrease by a factor of two during the search. Such an increase or decrease is linear with time and terminates after a characteristic time $d_{r}^{2}/2D$, so after that time $\gamma_{b}$ and $\gamma_{d}$ will remain constant for the rest of the simulation. Solid and dashed lines correspond to constraints in the workforce available $N_{w}=0.05N_{col}$ and $N_{w}=0.2N_{col}$, respectively, meaning that only regions on the left of such lines are accessible, provided that only 5% or 20% of the colony individuals can get engaged in foraging tasks. The maximum value of $\Delta E^{*}$ for such regions is indicated with an ‘x’ (note that for some cases this point lies in a region where ΔE does not change significantly, and so the heatmap shows a relatively constant colour).

Inspired by our experimental results (increasing $\gamma_{b}$ and decreasing $\gamma_{d}$ trend over time), we also analysed how time-dependent departure and return rates affected the energy-balance parameter space. Specifically, we allowed either $\gamma_{b}$ or $\gamma_{d}$ to increase (or decrease) from an initial value, denoted as $\gamma_{b}(t=0)$ and $\gamma_{d}(t=0)$, until reaching twice (or half) that value at time $t=d_{r}^{2}/2v^{2}\Delta t$, which is on the order of the expected search time required to find a food item located at distance $d_{r}$. After this point, the rates were kept constant at the values attained. This procedure allowed us to explore how gradual adjustments in departure and returning rates, including those consistent with the temporal patterns observed experimentally, reshape the colony’s energetic balance landscape. We stress that this choice for increasing or decreasing the rates $\gamma_{b}$, $\gamma_{d}$ is based on typical experimental variations observed in Fig 1, but it is somewhat arbitrary; for this reason, we have checked that using different protocols (using, for instance, three-fold increases or decreases) would not modify our conclusions.

We observe that more favourable energetic situations—that is, higher values of ($\Delta E^{*}$), consistently cluster in the region of large $\gamma_{b}$ and small $\gamma_{d}$. Accordingly, Fig 2 (right) shows that either increasing $\gamma_{b}$ or decreasing $\gamma_{d}$ leads to more favourable values of $\Delta E^{*}$ compared with the reference case (large panel on the left) where both rates remain constant. Conversely, increasing $\gamma_{d}$ or decreasing $\gamma_{b}$ shifts the system toward less favourable energetic outcomes.

In the Supplementary Information File S1 Text we show that, according to the energy balance in Eq. 5, large workforce loads and synchronous individual searches reduce first-passage times $T_{fp}$ while keeping collective search costs relatively high. Nonetheless, in resource-limited scenarios, where resources are far away or difficult to detect, finding and collecting the food takes more time and the collective search cost $T_{C}$ of many synchronous search trajectories becomes too high and might be reduced exploiting more asynchronous search strategies, where ant workers depart the nest at slower rates. The fact that, in our simulations, the most favourable energetic balances (Fig 2) are obtained for large $\gamma_{b}$ and small $\gamma_{d}$ simply reflects that the search time $T_{fp}$ dominates the energetic balance over $T_{C}$. In our search initial condition, engaging an increasingly large number of individuals in the search process would be the optimal strategy.

While predation, environmental disturbances, and other detrimental factors are not explicitly modelled, their net effect of reducing the number of active foragers can be implicitly captured by the maximum workforce parameter ($N_{w}$), which represents an upper bound on the number of ants available for sustained collective foraging. Given the condition ($\gamma_{b}/\gamma_{d}\leq N_{w}$), this is equivalent to imposing an upper bound on the ratio $\gamma_{b}/\gamma_{d}$. In the heatmaps, we therefore indicate the region of $\gamma_{b}$ and $\gamma_{d}$ values available to the colony under the assumption that only a fraction ($N_{w}/N_{col}$) of individuals can be engaged in foraging. These accessible regions correspond to the areas on the left of the solid and dashed lines for the cases ($N_{w}/N_{col}=0.05$) and ($N_{w}/N_{col}=0.2$), respectively.

The interpretation is straightforward: if, for example, only (5%) of the colony can be allocated to foraging—due to risks or because the remaining workers must perform other tasks—then only the region to the left of the solid line is attainable. Within this constraint, the colony would maximise its fitness by occupying a point in that region where $\Delta E^{*}>0$. For completeness, we mark in the heatmaps the specific positions where ($\Delta E^{*}$) reaches its maximum under the $N_{w}$ restriction (denoted by “x” symbols). Notably, these optima typically occur at nontrivial intermediate values of $\gamma_{b}$ and $\gamma_{d}$.

### 2.3. Energetic balance as a function of the relative workforce and colony size

Next, we analyze in more depth whether the availability of the workforce $N_{w}$ constrains the potential foraging activity of a colony and its energetic balance. First, in Fig 3 we show the maximum energy balance $\Delta E_{X}^{*}$ (this is, the energy balance reached at the ‘x’ points of Fig 2) as a function of the foraging workforce available ($N_{w}$) for colonies of different sizes ($N_{col}=100$ and $N_{col}=500$, corresponding to small and large circles in the plots, respectively). We compare the reference case ($\gamma_{b}$ and $\gamma_{d}$ constant, empty circles) to the more favourable case corresponding to ‘increasing $\gamma_{b}$’ (full circles), where the latter, as expected, reaches always higher $\Delta E_{X}^{*}$ values.

**Fig 3:**
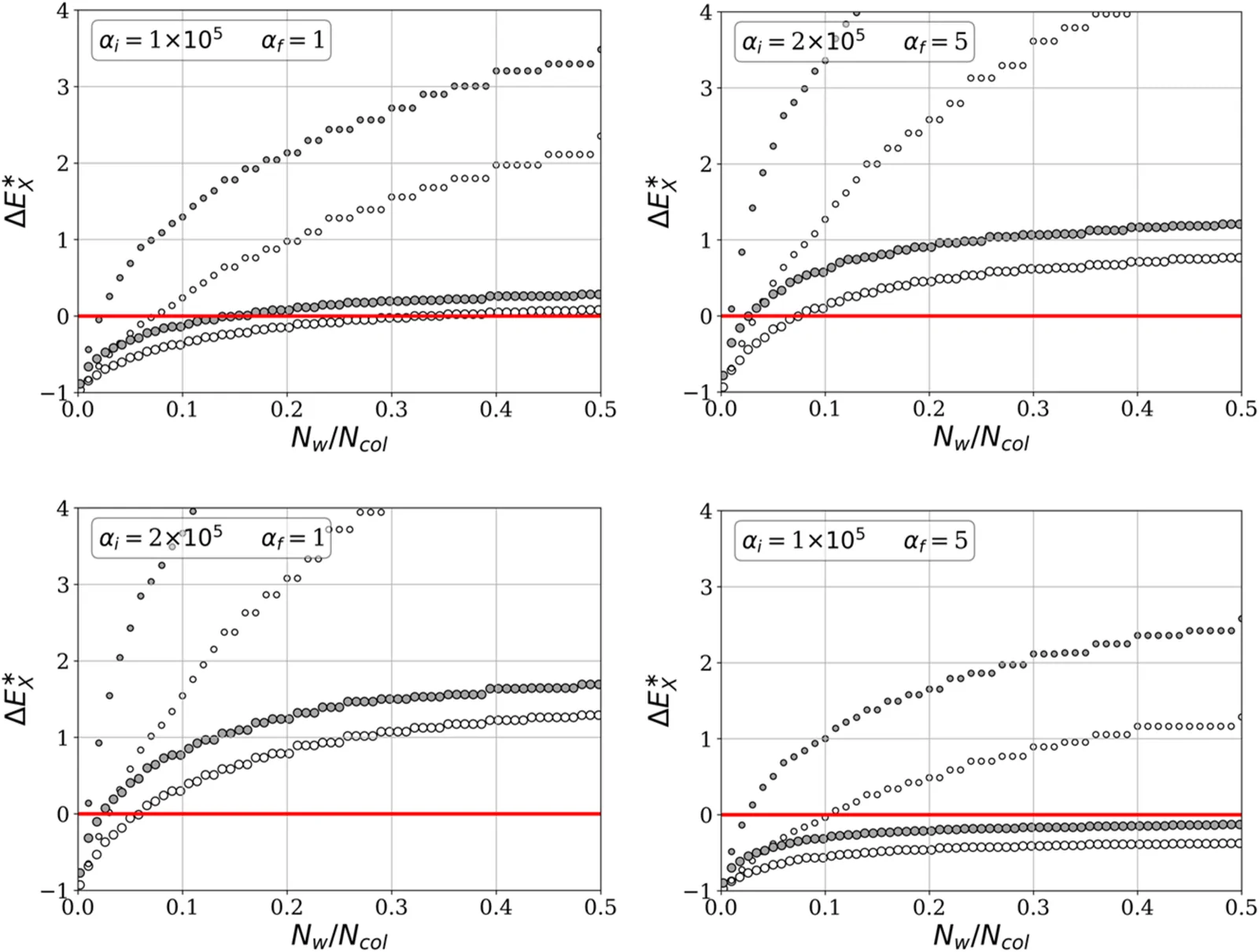
Maximum energy balance that can be reached by a colony of size $N_{col}=100$ (small circles) and $N_{col}=500$ (large circles) as a function of the foraging workforce $N_{w}$ (expressed as a fraction of $N_{col}$). Empty circles represent the cases with $\gamma_{b}$ and $\gamma_{d}$ constant, and black circles show the case with increasing $\gamma_{b}$. Different values of $\alpha_{i}$ and $\alpha_{f}$ are considered (indicated in the plot legends).

Fig 3 shows consistently (i.e., for different choices of $\alpha_{i}$ and $\alpha_{f}$ values) that for a small colony under the same search conditions, foraging becomes much more cost-effective. The viability of the large colony is always compromised in comparison to that of the small colony, as $\Delta E_{X}^{*}$ values for the former are always lower. Actually, we identify situations (see case $\alpha_{i}=10^{5}$, $\alpha_{f}=5$) where the large colony is not viable (i.e., $\Delta E_{X}^{*}<0$ for any workforce $N_{w}$), while the small colony is.

Even in cases where both colonies can achieve a positive energetic balance, there is always a minimum workforce $N_{w}$ required for such energetic viability to be reached. This highlights that having a sufficiently large workforce represents a significant constraint for these colonies. Moreover, this requirement becomes increasingly restrictive as colony size grows. This can be seen by noting that the minimum workforce needed to obtain a positive energy balance is smaller for the case $N_{col}=100$. In conclusion, small colonies appear to be less limited by the amount of workforce required to collect food in an energetically efficient manner.

The model omits several colonial life history traits that are likely to modulate foraging dynamics in natural populations, including worker turnover rate and experience, colony lifespan, foraging range, and tolerance to environmental stress. These factors may help explain how colonies persist and grow despite apparent limiting factors in collective foraging, like the relative numbers of active workers compared to colony size ($N_{w}/N_{col}$). The observation from our experiments is that small colonies tend to commit a larger proportion of their workforce to active foraging suggests a compensatory strategy, but the mechanisms determining the minimal number of active workers required, whether driven by nutritional stress, worker experience, or demographic constraints, remain unaccounted for in the current framework.

To complete our understanding of how energetic balances constrain ant colony foraging processes, Fig 4 shows how the optimal values of $\gamma_{b}$, $\gamma_{d}$ (those reached at the ‘x’ points, denoted by $(\gamma_{b}{)}_{X}$ and $(\gamma_{d}{)}_{X}$) vary with the colony size and the available workforce.

**Fig 4:**
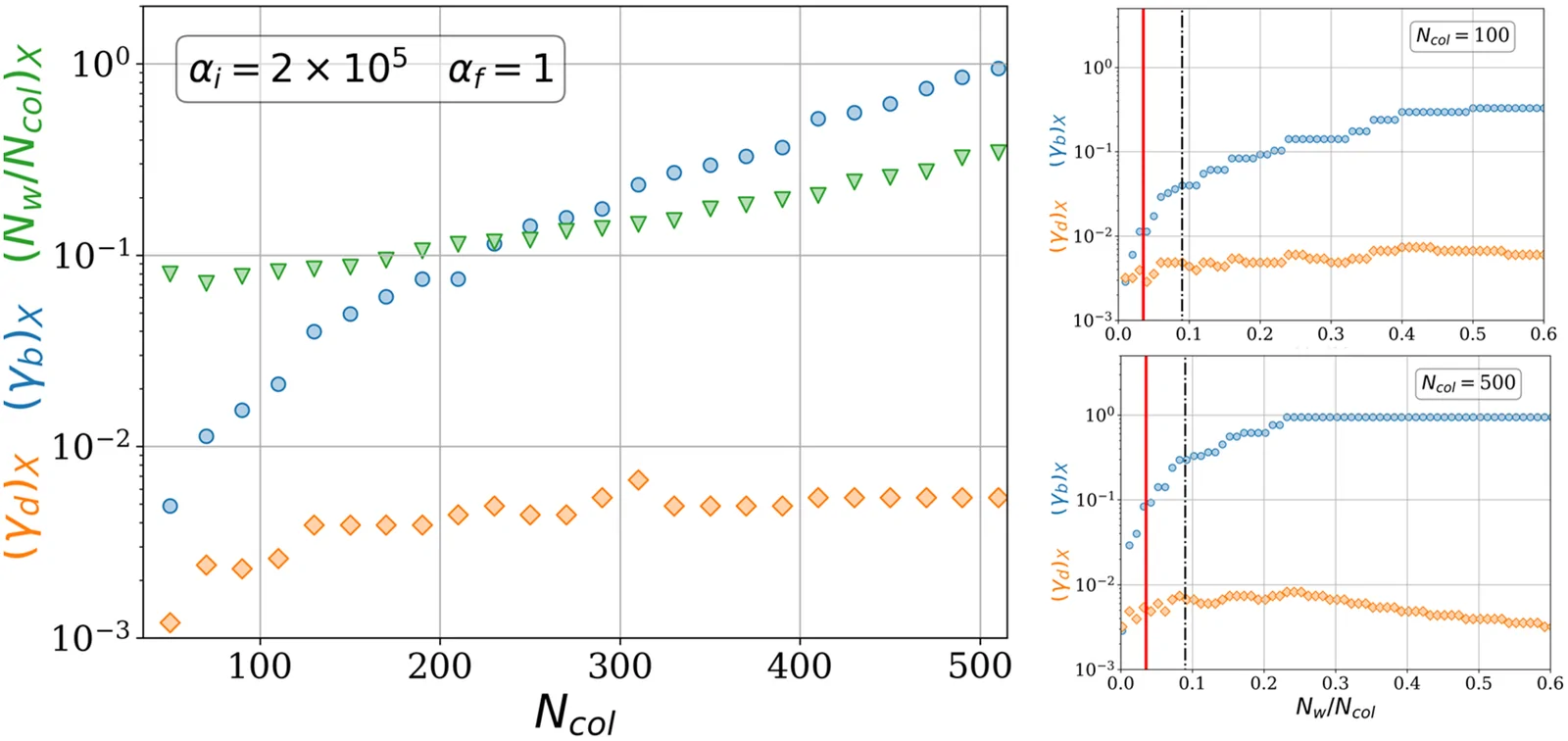
Left: Rates $\gamma_{b}$ (blue circles) and $\gamma_{d}$ (orange diamonds) corresponding to the maximum energy balance $\Delta E_{X}^{*}$ as a function of the colony size. The relative workforce $N_{w}/N_{col}$ corresponding to that optimal balance in every case is also shown (green triangles). Right: The same rates $\gamma_{b}$ and $\gamma_{d}$ are plotted for two colony sizes $N_{col}=100$ and $N_{col}=500$ (see legend) as a function of the foraging workforce $N_{w}$ (expressed as a fraction of $N_{col}$). The vertical red solid line identifies the minimum workforce corresponding to $\Delta E_{X}^{*}=0$, while the black dashdot line identifies the optimal workforce value reached when the energy balance reaches a maximum ($\Delta E_{X}^{*}=MAX$).

As colonies grow larger, achieving $\Delta E_{X}^{*}>0$ requires considerably larger departure rates. This leads to $\gamma_{b}$ (blue circles) spanning a wide range of values (varying by nearly two orders of magnitude); instead, $\gamma_{d}$ (red diamonds) remains comparatively stable, typically changing by less than a factor of two. This confirms again that the workforce load required to reach the maximum positive energetic balance increases with colony size. In practical terms, larger colonies need proportionally larger workforces (green triangles) to remain viable, and this increase in active foragers arises primarily from higher rates of recruitment from the nest, rather than from longer foraging trajectories.

Fig 4 (right panel) also compares the two colony sizes, large ($N_{col}=500$) and small ($N_{col}=100$)), and examine how increasing the workforce load ($N_{w}/N_{col}$) influences the optimal departure and return rates. Here, vertical lines are included as a visual guide to mark the threshold where $\Delta E_{X}^{*}<0$ (solid red line), and the point where $\Delta E_{X}^{*}$ reaches its maximum value (dashed black line). Once again, these results show that engaging a larger workforce in the foraging process is achieved primarily by increasing $\gamma_{b}$, that is, by launching new search trajectories at a higher rate. It is also noteworthy that $\gamma_{d}$ consistently reaches a maximum and then declines once $N_{w}/N_{col}$ becomes sufficiently large (values above 0.45 for $N_{col}=100$, and above 0.25 for $N_{col}=500$). In these specific regions, the colony increases the number of active searchers by extending the duration of their search trajectories (decreasing the return rates) rather than by launching new ones. In small colonies ($N_{col}=100$), however, this shift is much milder and occurs only at very high (likely unrealistic) values of $N_{w}$. Within the more relevant region between the minimum ($\Delta E_{X}^{*}<0$; solid red line) and maximum ($\Delta E_{X}^{*}=MAX$; dashed black line) energetic balances, variation in $(\gamma_{b}{)}_{X}$ remains the dominant mechanism driving changes in workforce engagement, whereas adjustments in $(\gamma_{d}{)}_{X}$ play a comparatively minor role.

## 3. Discussion

While there is substantial evidence that eusocial colonies can adjust their behaviour in response to environmental perturbations, most documented cases focus on traits such as nest-site selection or resource exploitation. The specific modulation of search patterns through workforce load variation has received comparatively little attention.

Our model addresses this gap but does not capture the full complexity of factors that may influence search efficiency in natural colonies, including behavioural plasticity, worker experience, or key life history traits. Nevertheless, a central contribution of this framework is to generate concrete, testable predictions about how the balance between active (i.e., high-metabolic-cost) foragers and inactive (i.e., low-metabolic cost) workers shapes collective search efficiency. By making this trade-off explicit, the model provides a comparative baseline against which the contribution of other biological factors can be evaluated, and opens the way for empirical tests of how colonies of different sizes regulate their active foraging fraction under natural conditions.

The metabolic rate of a colony is the aggregate of the metabolic rates of its individual members. When these individual rates are heterogeneous, one potential mechanism for regulating overall energy consumption is to adjust the relative proportions of high- and low-metabolism workers. In our framework, we capture this in its simplest form by distinguishing two metabolic states: active foragers, engaged in costly search behaviour outside the nest, and the rest, whether occupied with nest-bound tasks or waiting in the arena to be recruited. Accordingly, colonies face nontrivial trade-offs in determining how much workforce they can allocate to foraging. These trade-offs constrain both the potential rate of energy intake and the range of positive energetic balances that can be sustained as colonies increase in size. Together, these factors suggest that metabolic heterogeneity play a central role in shaping how colonies manage their energetic budgets during growth.

Our results reveal key behavioural adaptations that eusocial ant colonies may use to regulate their workforce $N_{w}$ through the rates of worker departure ($\gamma_{b}$) and return ($\gamma_{d}$) from and to the nest. Within our modelling framework, we can hypothesize how a colony might adapt to an environment characterised by specific values of $\alpha_{i}$ and $\alpha_{f}$. When these parameters make it relatively easy to maintain a positive energetic balance, colony fitness, and consequently colony size would increase. However, as colonies grow, they face progressively tighter constraints on the feasible range of workers (i.e., $N_{w}$ value) they can deploy. In other words, environments that allow colonies to thrive may simultaneously impose stricter limits on the proportion of workers that can be continuously engaged in active foraging as colony size increases. While small colonies can remain viable by deploying only a modest number of active searchers, larger colonies face a greater energetic burden per forager, making a high fixed proportion of active foragers increasingly costly to sustain. This scaling effect suggests that, for large colonies, maintaining a reserve workforce, a pool of inactive workers that can be facultatively recruited into foraging tasks when needed, becomes an advantageous, perhaps even necessary, evolutionary strategy. Rather than committing a large fixed fraction of workers to continuous foraging, dynamic recruitment from this reserve allows larger colonies to buffer energetic costs while preserving responsiveness to environmental demand.

In this context, our results provide further support for the idea that the presence of “lazy” or “inactive” workers [15–21], that is, individuals not heavily engaged in any specific task, should not be interpreted as inefficiency. Instead, they constitute a mechanism that maintains a permanent pool of workers ready to be mobilised when the colony’s energetic demands increase. This interpretation aligns with explanations for the sublinear scaling of metabolic rates observed in eusocial colonies and other social groups [19,45–47]. According to this view, larger colonies favour a higher proportion of inactive workers, or equivalently, impose limits on the effective workforce load (from low- to high-metabolism individuals) as colony size increases. Empirical studies support this pattern: artificially increasing colony size leads to a higher proportion of inactive individuals [48], and in some species these can represent more than 50% of colony members [18,23].

These observations are not incompatible with the fact that larger colonies may enjoy competitive advantages — including access to broader foraging ranges, greater territorial dominance, and more efficient resource recruitment — that offset their higher absolute metabolic costs [49–51]. What matters, under this view, is not total resource intake but the net energetic balance at the colony level.

Ecological context further shapes how dynamic workforce modulation may benefit search efficiency in colonies. The spatial distribution of resources such as their average distance from the nest, degree of clustering, and the structural complexity of the habitat, is a major constraint on collective exploration and foraging efficiency [52]. Our results predict that environments where food detection is difficult (i.e., where energy intake $E_{intake}$ or, equivalently $\alpha_{i}$, is reduced) should select for lower $\gamma_{b}$ values and potentially higher $\gamma_{d}$ values, resulting in a smaller available workforce $N_{w}$. Colonies adapted to such low-resource habitats would therefore tend to remain small, relying on search strategies characterised by fewer, more asynchronous, and longer foraging trajectories. In contrast, habitats with higher resource availability should allow colonies to expand in size and adopt search strategies involving a larger number of synchronous foragers performing shorter trips.

Most empirical studies examining the relationship between foraging activity and resource availability in eusocial species report patterns consistent with our predictions. Low resource density is typically associated with fewer but longer foraging trajectories, as well as a negative correlation between the distances travelled by searchers and the degree of group recruitment [11,12,53,54]. Notably, studies such as [33,55] have shown that these effects are largely independent of group size, supporting the idea that maintaining a reserve workforce can stabilize exploration strategies across a range ofenvironmental conditions. In contrast, small groups of non-eusocial species (e.g., rodents) often exhibit group size-sensitive foraging patterns [56], highlighting a distinct collective dynamics than that in eusocial systems. Interpreting these results, however, is not always straightforward, as many reported foraging behaviours include phases of resource exploitation or are conducted under conditions where colonies may have been previously conditioned by prior knowledge of resource locations.

The effect that movement/navigation strategies may have is also nontrivial. As long as search trajectories become less random and more systematic (e.g., Archimedean spirals or similar patterns exhibited by some animals), then multiple search trajectories would be less and less necessary (as one single trajectory is enough for covering perfectly the whole domain, or a significant part of it). Then, optimal strategies would shift to low $\gamma_{b}$, low $\gamma_{d}$. Indeed, for species using chemical cues, landmarks, or other cognitive shortcuts that reduce uncertainty or stochasticity, we would similarly expect to see a similar trend. This is actually another prediction from our model, the idea that species which rely more on external cues for searching will exhibit more ‘asynchronous’ search patterns than those lacking this ability or those adapted to more uncertain/variable conditions.

If we assume that collective cognitive programs are themselves subject to selection, a view that is increasingly supported in the ecological literature [15,57–61], then our results offer a natural explanation for (i) the existence and functional relevance of inactive workers in large eusocial colonies, and (ii) the resulting metabolic scaling of colonies with their size. Together, these insights suggest that functional redundancy and the maintenance of workforce reserves are not incidental features of eusocial societies, but key components of their energetic stability, resilience, and evolutionary success.

More broadly, we argue that the redundancy principle [24] provides a valuable lens for understanding collective behaviour in ecological systems. This principle may operate across multiple tasks within the colony (not only in exploration), which would help explain the strong selection observed for the presence of task-unengaged individuals. Long-term monitoring of task allocation within colonies [62,63], combined with controlled perturbations that disrupt one or several tasks and analysed within an energetic-cost framework such as the one developed here, would offer a powerful approach to testing this possibility.

## 4. Methods

### 4.1. Empirical data acquisition and processing

In order to explore how ant colonies arrange and regulate workforces during a search, we used experimental data obtained in the lab (under constant temperature conditions at 20ºC) from two different experiments, each conducted with two different colonies of *Aphaenogaster senilis* ants.

**Experiment A.** First, we conducted an experiment in an arena consisting of a set of consecutive parallel (leftwards and rightwards) lines, so reproducing a quasi-1d topological space (see Fig 5a). The nest was connected through a plastic tube to one end of the structure, and the lines were surrounded by water to prevent the ants to escape, or get out from the structure. Food (consisting of small amounts of cookies and egg yolk) was located 1 meter away from the nest entrance. Then, three different food conditionings were considered by either adding new food items at 2 meters from the nest (high-density condition), at 3 meters away from the nest (medium-density condition), or non adding any additional resources (low-density condition).

**Fig 5:**
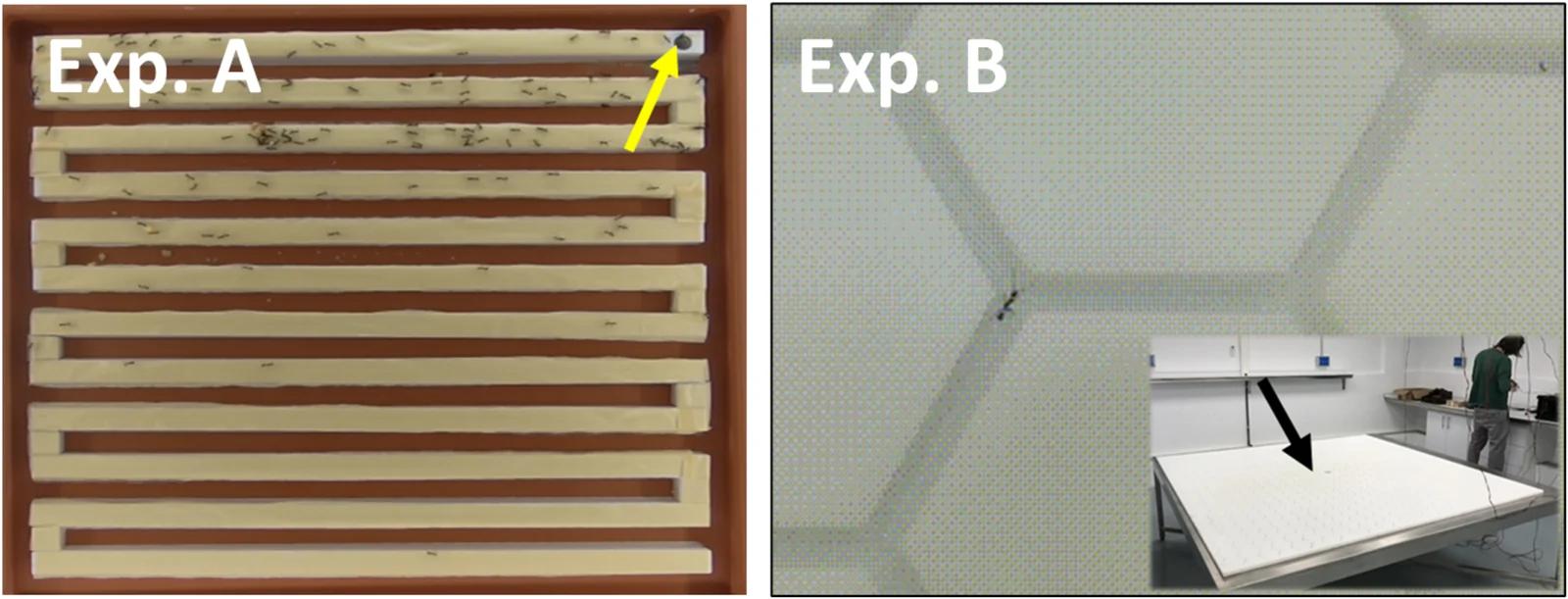
Illustration of the different arenas used to obtain the experimental data used in the work (the arrows identify the entrance to the nest in each case). Experiment A: Quasi-1d arena consisting of parallel lines leftwards and rightwards. Experiment B: Hexagonal lattice (the main image shows a close-up of the lattice, while the inset on the lower right corner corresponds to the whole structure).

In this experiment we compared the foraging dynamics of two colonies of different size (designated colonies A1 and A2 in the following). Colony A1 had $N_{col}=200$ individuals and colony A2 $N_{col}=60$ approximately (these numbers did not vary significantly during the experiments). Three experiments per week (in nonconsecutive days) were conducted for each colony until completing a total of five experiments for each colony and each food conditioning.

**Experiment B.** Additional experimental datasets were obtained from a previous setup [44] corresponding to two other colonies of approximately the same size ($N_{col}\approx 750$ individuals), named B1 and B2. These were allowed to explore a large arena consisting of a hexagonal lattice with a total size of 2x1 meters (see Fig 5b). The details about this second setup and dataset have already been given and discussed in [44]. Three food conditions were considered for this case: (i) deterministic, (ii) stochastic, and (iii) no-food. In the deterministic case food resources were located at two nodes (always the same) in the lattice at a medium distance from the nest, while in the stochastic case the resources were located at two different nodes selected at random every day.

In both experiments A and B the foraging process (starting when the arena is connected to the nest) was recorded in video for a period of 2 hours, which was far enough for the ants to find and collect the food, and to explore the rest of the arena. Actually, the period of food gathering typically elapsed about 30–50 minutes. The recording was done at a frequency of 10 Hz, and the analysis of the frames was carried out with standard libraries (in Matlab). As a result, we could compute the number of active ants present in the arena during the whole foraging process, $N_{a}(t)$.

From the data for $N_{a}(t)$ we could estimate average values of the rates $\gamma_{b}$ and $\gamma_{d}$. For this, we considered a moving time window of size τ throughout the experiments, and we computed the number of search trajectories appearing and terminating within the window ($n_{in}$ and $n_{out}$, respectively). According to the definition above, the average rate at which search trajectories appear is given by $\gamma_{b}=n_{in}/\tau$. Similarly, for all the ongoing trajectories present in the arena we have that they can be terminated at a rate $\gamma_{d}$ according to our definition, so we use $\gamma_{d}=n_{out}/\langle N_{a}\rangle_{\tau }\tau$ (where $\langle N_{a}\rangle_{\tau }$ represents the number of active ants in the arena averaged over the time window τ). At practice, we took τ=10 min, which provided enough events (appearing and terminating trajectories) within each window for reliable rate estimates while remaining short enough to let us observe an evolution of the foraging strategies through time.

### 4.2. Computational model and parameters

We used a stochastic search model where searchers carry out random walks on an infinite plane starting from a common point (representing the nest). At time *t* = 0 a first search trajectory starts from the nest, and subsequently new random-walk trajectories appear (disappear) at rates $\gamma_{b}$ ($\gamma_{d}$), respectively. The corresponding search process is kept until the resources, located at a single random patch at a distance $d_{r}$ from the nest, are hit by a trajectory (this happens if the distance from the trajectory to the resources is lower than a detection distance $d_{d}$).

For simplicity, we assume that random walks obey classical Brownian motion, so each trajectory consists of a combination of independent Gaussian jumps with a characteristic speed *v* in random uncorrelated directions. Then, the length covered in a single jump during a time step Δt is vΔt. Accordingly, Δt represents the characteristic directional persistence time of the trajectories, so for times shorter than Δt the motion is persistent, while for times much longer than Δt the direction of motion becomes uncorrelated (see [64] for a comprehensive discussion on the topic). We will focus in the low-persistence regime given by $\Delta t\ll d_{r}/v$, which at practice means that a large number of jumps is required to cover the distance $d_{r}$ from the nest to the target, and so diffusive motion will dominate the relevant timescales for the search process.

In summary, the algorithm of the model used takes the form

For each time step t→t+Δt, do:

i) *With probability* $\gamma_{b}\Delta t$ *a new trajectory is initiated at the nest position.*
ii) *For every present foraging trajectory:*
  iia) *With probability* $\gamma_{d}\Delta t$*, terminate the search trajectory.*
  iib) *With probability* $1-\gamma_{d}\Delta t$*, add a new Gaussian displacement (with standard deviation* vΔt*) to the present position of the trajectory.*
  iic) *If the resulting position is within a distance* $d_{d}$ *to the resource location, then terminate the search.*

Note that the number of simultaneous search trajectories in the system will change with time. If we denote this number by $N_{a}(t)$, our initial condition states that $N_{a}(0)=1$, and then $N_{a}(t)$ will either increase or decrease until reaching the stationary value $\gamma_{b}/\gamma_{d}$ on average. At practice, for $\gamma_{b}\gg \gamma_{d}$ the number of searchers will increase to reach $N_{a}(t)=\gamma_{b}/\gamma_{d}$ and then it will fluctuate around this value. Then, by denoting the number of ants in the colony as $N_{col}$, and assuming that only a part $N_{w}<N_{col}$ of them is susceptible to become recruited for foraging, the condition $\gamma_{b}/\gamma_{d}\leq N_{w}$ must be satisfied, which sets a limitation on the range of $\gamma_{b}$, $\gamma_{d}$ values that are accessible to the colony.

For the contrary case, $\gamma_{b}\ll \gamma_{d}$, most of the time we will have either just one single search trajectory ($N_{a}(t)=1$) or the search space will be empty ($N_{a}(t)=0$), and so the colony will rely on an *individual* foraging strategy.

As an output of the model, we computed the Search Time $T_{fp}$ (this is, a first passage time, the time it takes for at least one of the trajectories to hit the resources for the first time), and the Collective Time Cost $T_{C}$, defined as the combined duration of all trajectories that have emerged from the nest up to time $T_{fp}$.

### 4.3. Estimation of the energy balance in the colony

Since the main objective of the model above is to estimate whether an increasing workforce $N_{w}$ available is beneficial or not for the colony, we need to estimate the energy intake and costs related to the search. The question then is how to quantify this effect, this is, how to define a proper fitness function or measure that the ants are trying to increase/maximize [65]. For the context considered in our work, we will assume that colonies should try to reach a positive balance between their energy consumption (which includes the basal consumption and the energy cost of foraging) and the energy intake obtained through foraging.

In the following, we describe how the energy balance can be conveniently written in terms of the parameters $N_{col}$, $N_{w}$, $T_{fp}$ and $T_{C}$ introduced above. For this, we assume that ants in the colony have always a constant basal metabolic rate ${\text{RM}}_{b}$, and active foragers have an additional working metabolic rate ${\text{RM}}_{a}$, so active foragers have a total metabolic rate ${\text{RM}}_{b}+{\text{RM}}_{a}$. Also, we introduce $T_{f}$ as the daily time that the colony spends foraging (so $T_{f}<T_{day}$, with $T_{day}$ representing the total duration of one day). Given our definition for the time required to find a food item, $T_{fp}$, the average number of items that can be obtained by the colony in a single day is then $T_{f}/\langle T_{fp}\rangle$ (we use the bracket notation ⟨·⟩ to denote averages over the random trajectories, and $\langle \cdot \rangle_{t}$ to denote time averages, so $\langle N_{a}\rangle$ is the mean number of active workers at a given time averaged over different realizations of the random-walk simulation, and $\langle N_{a}\rangle_{t}$ is the average of active workers over time for a single simulation).

Taking into account all these considerations, the different terms of the energy balance read:

1. **Energy intake.** If we denote the energy content of every food item as $E_{item}$, the daily intake obtained by the colony as a result of the foraging process is given by

$$\begin{array}{c} E_{intake}=E_{item}\frac{T_{f}}{\langle T_{fp}\rangle }, \end{array}$$ (1)

where we are not explicitly considering the gathering time (that is, the time it takes to bring the food items to the nest), but only the time required to find the food. We do so because for most ant species such gathering time, while can be long, typically overlaps with new exploration trajectories, so we do not need to include it explicitly in the intake energy. Additionally, we are assuming that ant colonies communicate information fast enough upon food detection, so recruitment becomes activated immediately to exploit the resource (our experimental datasets confirm this approximation is quite reasonable, as in the conditions used food detection takes typically 15–40 minutes, while recruitment seems to happen always in less than 5 minutes).

2. **Basal consumption.** According to the definitions above, the basal consumption for the whole colony of size $N_{col}$ will be denoted by

$$\begin{array}{c} E_{basal}=N_{col}T_{day}{\text{RM}}_{b}. \end{array}$$ (2)

3. **Foraging cost.** The foraging cost will take into account the average number of active workers, $N_{a}$, during the foraging time $T_{f}$. So that, it can be written as

$$\begin{array}{c} E_{foraging}=\langle N_{a}\rangle_{t}T_{f}{\text{RM}}_{a}=\langle T_{C}\rangle \frac{T_{f}}{\langle T_{fp}\rangle }{\text{RM}}_{a}, \end{array}$$ (3)

according to the definition of $T_{C}$ given above, from which $\langle T_{C}\rangle =\langle N_{a}T_{fp}\rangle_{t}\approx \langle N_{a}\rangle_{t}\langle T_{fp}\rangle$ follows (this is, we take the time average of the product as the product of individual averages as if these two magnitudes were independent. Note that the time average of $T_{fp}$ is ill-defined, as $T_{fp}$ is itself a time, so an average over realizations must be used instead).

In consequence, the energy balance for the colony takes the form


$$\begin{array}{c} \Delta E=E_{intake}-E_{foraging}-E_{basal}, \end{array}$$ (4)

which can be conveniently rewritten as


$$\begin{array}{c} \Delta E^{*}\equiv \frac{\Delta E}{E_{basal}}=\alpha_{i}\frac{1}{N_{col}\langle T_{fp}\rangle }-\alpha_{f}\frac{\langle T_{C}\rangle }{N_{col}\langle T_{fp}\rangle }-1, \end{array}$$ (5)

after introducing the parameters $\alpha_{i}\equiv E_{item}T_{f}/(T_{day}{\text{RM}}_{b}$) and $\alpha_{f}\equiv T_{f}{\text{RM}}_{a}/(T_{day}{\text{RM}}_{b}$). These parameters $\alpha_{i}$ and $\alpha_{f}$ then represent relative *intake* gain and *foraging* cost, respectively. Since the metabolic rates and the rest of parameters involved in their definition are case and species specific, and often they are not available experimentally, here we will rather explore the behavior of $\Delta E^{*}$ (by running random-walk simulations to compute search times) as a function of $\alpha_{i}$ and $\alpha_{f}$, with the focus put on identifying the region where the balance $\Delta E^{*}$ becomes positive, which determines the condition for the colony to become energetically viable.

In particular, the questions we will address in the present work are: given some values of $\alpha_{i}$ and $\alpha_{f}$, what are the combinations of $\gamma_{b}$, $\gamma_{d}$, $N_{col}$, $N_{w}$ values (with the aforementioned constraint $\gamma_{b}/\gamma_{d}\leq N_{w}$) that lead to $\Delta E^{*}>0$? When is it convenient for the colony to increase its workforce $N_{w}$ as a mechanism to increase its energy intake, even at the expense of increasing its foraging cost?

## Supporting information

S1 Text File S1 text contains additional analysis/results of the experimental and computational datasets, including severa Figures (A to D). (ZIP)
