# Supplementary material for: Dynamic workforce modulation and foraging efficiency in Eusocial insect colonies
Source: PLoS Comput Biol. 2026 Oct 9;22(10):e1014258. doi: 10.1371/journal.pcbi.1014258 (PMC13667929; doi:10.1371/journal.pcbi.1014258)
Supplement: Supplementary file 1 [file pcbi.1014258.s002.pdf]

**Title:** Dynamic Workforce Modulation and Foraging Efficiency in Eusocial Insect Colonies
**Authors:** D.Campos, J.Cristin , P.Fernandez-Lopez and F.Bartumeus

The authors study both experimentally and theoretically how the foraging dynamics of ant colonies depends on the availability of a redundancy workforce that can be activated depending on environmental conditions. A stochastic energy-balance framework is developed coupled to the availability of foraging ants, which is studied in computer simulations. Results are qualitatively compared to experimental findings.

I find the topic of this ms. very timely and interesting, especially as from the theoretical side studying foraging success depending on the number of foragers attracted quite some attention recently. Overall the ms. is very well written, even though I have some remarks especially on the introduction. The article reports novel experimental results, see Fig.3, that are to some extent supported by simulations obtained from solving a stochastic model, see the other figures, which provides a nice mix between theory and experiment. The present work partially builds on the previous brief Ref.[40] by two of the authors, however, apart from inspiration regarding the stochastic model there is no significant overlap.

In summary, I enjoyed reading through this ms., and I expect it to be publishable in PLoS Comp. Biol. after my minor remarks below are suitably taken into account.

1. abstract: Perhaps explicitly mention somewhere that ants are studied, to the not so well versed reader. And I had to google what eusocial means.

2. introduction: From the introduction it did not become very clear to me whether this is purely theoretical work or whether it includes experiments. In l58 the authors refer in passing to 'numerical and empirical evidence', but empirical could also have meant taking data from experiments published in other work. They may wish to make more clear that they do report new experimental results analysed within a theoretical framework by simulations of a stochastic model, as this is a strong point of the ms.

3. literature: On l65ff some theoretical work on 'diffusive individuals' is mentioned (Refs.[34-39]). But diffusion can be generated by very many different stochastic processes, so perhaps more details on the types of stochastic processes considered could be included. Also, studying the number dependence of stochastic search and first passage problems seems to have become a topic of quite some recent interest. There are, e.g., new recent preprints by Lawley (arXiv:2604.10004 2026) and Grebenkov et al. (arXiv:2602.15627). So perhaps the authors' citations about this topic need to be updated / amended respectively by doing another literature search.

4. l74: The previous Ref.[40] by two of the authors is explicitly referred to here but not much discussed anymore in the following. So none of the predictions

made in this previous paper could be tested by these two experiments? Is this so because therein the assumption of $\gamma_d = 0$ was made, which is not fulfilled here? While here it is stated that this previous work has also identified an optimal value for $\gamma_d$. Please check.

5. l81ff: The authors consider two experiments, where according to Fig.1 Exp.A is essentially one-dimensional. Nevertheless, in Fig.2 both experiments display qualitatively similar results. Can the authors comment on the expected impact of the dimensionality? See also Fig.6, where results seem to look a bit different, to some extent, though there may be other reasons. Furthermore, the stochastic model studied is only two-dimensional but still seems to somewhat work to qualitatively explain the one-dimensional results?

6. The list of references needs polishing. It is a bit inhomogeneous (some hyperlinks included but mostly not) and includes unspecified authors.
