# Supplementary material for: Dynamic workforce modulation and foraging efficiency in Eusocial insect colonies
Source: PLoS Comput Biol. 2026 Oct 9;22(10):e1014258. doi: 10.1371/journal.pcbi.1014258 (PMC13667929; doi:10.1371/journal.pcbi.1014258)
Supplement: Supplementary file 2 [file pcbi.1014258.s003.pdf]

l.11. There are no tests being conducted in the paper. Please rephrase.

l.67. Scales quadratically with dr would be a more transparent way to put it.

l.71 It is unclear at this point what (a)synchronous means. Please give a little more details.

l.83 *A. Senilis* is specialized towards arid environments, and thus shows very specific foraging strategies (fast lone scouts, visual navigation…). It would be useful that the authors discuss how this could have affected their findings (for instance in section l.348-357 of the discussion where this is mentionned). For instance, do you believe species with navigation strategies based on chemical cues (i.e., where exploration is more of a group endeavor) would yield qualitatively similar results?

l.113. What does "significant statistics" mean in that context?

l.115. It is unclear at this point what the computational model is going to be used for. It would be useful to have a small section at the end of the introduction that lays out the main steps of the analyses, and explains their logic. The section l.174-177 does this but only after methods, could the same information be given verbally ?

l.123. I do not understand the notions of "persistence" and "diffusive scaling" in this context, and a quick google search did not help. Please give more explanations. Alternatively, it seems to me that maybe these sentences could be removed entirely, since it is enough to know that food items are place far away from the nest.

l.143. This sentence is grammatically incorrect. More importantly, neither organisms nor groups maximize their fitness. The fitness quantity that is likely to be maximized in an evolving population is the growth rate of gene lineages. Depending on the system, this quantity sometimes aligns with individual fitness (number of offspring) or group productivity, but this sentence is misleading as is. The paper cited (ref 42) makes exactly that point, and it is more about which currency *we* can use as fitness proxies when studying a system, than about the true quantity that selection optimizes.

l.153, 165. It would be useful to see the mathematical definition of averages over random trajectories and time. I have a hard time understanding exactly how quantities in brackets where calculated.

l.159 Processing time is not considered either. This could be especially important in species such as *A. senilis*, that can sometimes rely on large food items. Please discuss.

l.168. Since the energy balance increases with α_i, I found it confusing that this parameter is referred to as a cost. If nothing prevents it, please modify.

l.170. species

l.180-219. This section shows how observations of lab colonies have yielded important qualitative information, and how this information was used to guide the modeling work (l.227 as well). I think it would be worth stating this early in the main text. As is, I sometimes had a hard time understanding the links between the experimental and mathematical aspects of the article.

FIG2. To give a better account of statistical power, error bars should give the confidence intervals associated to the computed averages, not standard deviations.

L.221 It remains unclear to me why simulations where used only to determine Tfp and Tc, before plugging these values in eq. 1-5 to determine energy balance. Surely, energy balance could be determined from simulation directly? This would yield values less dependent on approximations, and could even be used to include more complex processes. Surely I am missing something here, please briefly explain.

l.229. What is the motivation behind a two-fold change in parameters. Why not a larger or smaller change?

FIG3. To be honest, it is hard for me to detect differences between the different panels of this figure. Is it certain that a different scaling/cropping would not help making these differences more evident?

l.249. I do not see how assuming that the colony has access to a maximum workforce account for predation. Please explain.

l.251. typo: γb)and(γd).

l.274. It also highlights that the model is missing some aspects of colonial life history. What would be the mechanisms that let small colonies persist and grow in natural populations, and that are not accounted for here?

l.305. While the statement here is likely true, it corresponds only loosely to the results of the present paper, since the modeling here does not include behavioral plasticity. Please give more details as to how the present framework could be used to include these aspects.

l.309. What testable hypotheses emerge here for instance?

l.312. What are high- and low-metabolism workers? do you mean foraging vs nest-bound?

l.322 typo “,”

l.324-328. To me, these two last sentences contradict each other. If larger colonies are on a tight budget, they should be less able to maintain a stationary reserve workforce. What am I missing here?

l.341. Do you mean αi?

l.354. What is the “opposite pattern”?

l.358. Group selection is at best an unfortunate simplification, with no serious empirical or theoretical ground. The existence of coherent group-level behaviour (thus favouring every individual within it) can be explained fully by gene-level selection. Please rephrase.
