# Supplementary material for: Dynamic workforce modulation and foraging efficiency in Eusocial insect colonies
Source: PLoS Comput Biol. 2026 Oct 9;22(10):e1014258. doi: 10.1371/journal.pcbi.1014258 (PMC13667929; doi:10.1371/journal.pcbi.1014258)
Supplement: Supplementary file 3 [file pcbi.1014258.s005.pdf]

Reviewer #1;

The authors study both experimentally and theoretically how the foraging dynamics of ant colonies depends on the availability of a redundancy workforce that can be activated depending on environmental conditions. A stochastic energy-balance framework is developed coupled to the availability of foraging ants, which is studied in computer simulations. Results are qualitatively compared to experimental findings.

I find the topic of this ms. very timely and interesting, especially as from the theoretical side studying foraging success depending on the number of foragers attracted quite some attention recently. Overall the ms. is very well written, even though I have some remarks especially on the introduction. The article reports novel experimental results, see Fig.3, that are to some extent supported by simulations obtained from solving a stochastic model, see the other figures, which provides a nice mix between theory and experiment. The present work partially builds on the previous brief Ref.[40] by two of the authors, however, apart from inspiration regarding the stochastic model there is no significant overlap.

In summary, I enjoyed reading through this ms., and I expect it to be publishable in PLoS Comp. Biol. after my minor remarks below are suitably taken into account.

We thank the reviewer for such a positive response. In the following we try to address the minor remarks mentioned:

1. abstract: Perhaps explicitly mention somewhere that ants are studied, to the not so well versed reader. And I had to google what eusocial means.

We appreciate this comment that could certainly facilitate reading to a general audience.

Changes made: We have rephrased some sentences in the abstract (in blue) to facilitate understanding.

2. introduction: From the introduction it did not become very clear to me whether this is purely theoretical work or whether it includes experiments. In l58 the authors refer in passing to 'numerical and empirical evidence', but empirical could also have meant taking data from experiments published in other work. They may wish to make more clear that they do report new experimental results analysed within a theoretical framework by simulations of a stochastic model, as this is a strong point of the ms.

We again appreciate this comment.

Changes made: We have tried to rewrite part of the introduction being more systematic and explicit about the aims and methodologies used in the work.

3. literature: On l65ff some theoretical work on 'diffusive individuals' is mentioned (Refs.[34-39]). But diffusion can be generated by very many different stochastic processes, so perhaps more details on the types of stochastic processes considered could be included. Also, studying the number dependence of stochastic search and first passage problems seems to have become a topic of quite some recent interest. There are, e.g., new recent preprints by Lawley (arXiv:2604.10004 2026) and Grebenkov et al. (arXiv:2602.15627). So perhaps the authors' citations about this topic need to be updated / amended respectively by doing another literature search.

We admit that our selected references were chosen several months ago, while the literature on first-passage problems on collective searches is currently very active, so we thank the reviewer for stressing this point.

Changes made: After a search on recent literature, we have added References [40-42] (in the new version) to the bibliography, and these are cited when theoretical aspects of collective searches are discussed.

4. l74: The previous Ref. [40] by two of the authors is explicitly referred to here but not much discussed anymore in the following. So none of the predictions made in this previous paper could be tested by these two experiments? Is this so because therein the assumption of γd = 0 was made, which is not fulfilled here? While here it is stated that this previous work has also identified an optimal value for γd. Please check.

We appreciate this insightful comment about our previous work [40]. Regretfully, our previous results in the previous Ref. [40] cannot be tested with our datasets. The problem is twofold. First, as stated by the reviewer the assumption γd = 0 made there would make direct comparison to the ants' case very difficult (we have now a paper in preparation extending those theoretical results to arbitrary γd, but unfortunately this is not the only constraint). Second, our theoretical results hold for γb fixed, while our data show that real foraging patterns are clearly nonstationary (actually, this is one of the results we discuss in the work). This, together with additional technical aspects like interindividual variability and experiment design, make such a comparison impractical.

Changes made: We have checked and rephrased the sentences discussing our results in [40] to state clearly the connection to the present work.

5. l81ff: The authors consider two experiments, where according to Fig.1 Exp. A is essentially one-dimensional. Nevertheless, in Fig.2 both experiments display qualitatively similar results. Can the authors comment on the expected impact of the dimensionality? See also Fig.6, where results seem to look a bit different, to some extent, though there may be other reasons. Furthermore, the stochastic model studied is only two-dimensional but still seems to somewhat work to qualitatively explain the one-dimensional results?

According to our theoretical results in [40] (plus those we have from an extended version now in preparation) dimensionality does not affect qualitatively the results on the search efficiency of groups, but only quantitatively. While these theoretical results represent just an approximation to the experimental process (as discussed above), one should expect that Fig. 2 shows similar patterns in all cases (either 1d or 2d foraging), only shifting γd and γb values. That shift, however, will no be high in our case because colonies used in 2d foraging were quite larger (N=750) than those in the 1d experiments (N=60 and N=200). So, size effects compensate for this dimensionality issue. This choice is not fortuitous, but we typically choose colony sizes in such a way that typical foraging timescales are not very different to those in field conditions (typically 1-2 hours).

Changes made: We have included some additional comments in the Methods Section to clarify why we expect our 2d model to approximately work in one-dimensional conditions.

6. The list of references needs polishing. It is a bit inhomogeneous (some hyperlinks included but mostly not) and includes unspecified authors

Changes made: After including the new references suggested by all the reviewers, we have carefully checked the whole list.

Reviewer #2:

In this article the authors explore the links between high-level colonial exploration parameters, the distribution of resources in the environment, and colonial productivity. With simple and overall well-explained methodology, combining modeling with experimental work, they highlight some of the core trade-offs that constraint foraging in eusocial groups. The theoretical approach is sound, but should be refined in the future to account for important aspects of colonial life history, such as variance in individual behaviour, predation, resource handling, or individual interactions during foraging.

The main limitation of the current manuscript, in my opinion, lies in the lack of higher-level discussion of the derived results. Too little effort is put in discussing the limitations of the model and experimental set-up, which may participate in heavily biasing the results. A good example is the result according to which large colonies have a harder time keeping their energy balance positive. This somewhat contrasts with common knowledge about ant colonies, which often produce large surplus only at larger sizes (due to better competitivity, access to different resources, synergy during foraging…). A better characterization of the model's limits, in the face of the complexity and diversity of eusocial systems, is required for a better integration of the results.

We thank the referee for such insightful comments, and for the constructive suggestions below. We hope that the changes introduced in the new version of the manuscript (e.g., Discussion section) can satisfactorily provide such a higher-level discussion. Despite we acknowledge and make explicit these limitations, it is also true that the particular observation that large colonies may be more competitive in general and improve total resource intake is not incompatible with a larger energetic demand. What our model is indicating is that what matters is the net energetic balance of the colony, it is, how resource intake grows compared to metabolic costs of active foragers.

l.11. There are no tests being conducted in the paper. Please rephrase.

Changes made: We agree with the reviewer, and have rephrased this sentence accordingly.

l.67. Scales quadratically with dr would be a more transparent way to put it.

Changes made: We agree. We have introduced the change as stated by the reviewer

l.71 It is unclear at this point what (a)synchronous means. Please give a little more details.

Changes made: We have rephrased the sentence explicitly giving a notion of what 'asynchronous' means in the present context

l.83 A. Senilis is specialized towards arid environments, and thus shows very specific foraging strategies (fast lone scouts, visual navigation…). It would be useful that the authors discuss how this could have affected their findings (for instance in section l.348-357 of the discussion where this is mentioned). For instance, do you believe species with navigation strategies based on chemical cues (i.e., where exploration is more of a group endeavor) would yield qualitatively similar results?

We thank the reviewer for this comment; this is certainly a very interesting point of discussion. Our theoretical model assumes for simplicity that search trajectories are Brownian, but the effect that different navigation strategies may have is not trivial. What we can state is:

(i) As long as search trajectories become less random and more systematic (e.g. Archimedean spirals or similar patterns exhibited by some animals) then multiple search trajectories would be less and less necessary (as one single trajectory is enough for covering perfectly the whole domain, or a significant part of it). Then, optimal strategies would shift to (low γb - low γd) patterns.

(ii) Then, for species using chemical cues, landmarks, or other cognitive shortcuts that reduce the levels of stochasticity in search, we would expect to see a similar trend, with lower rates γb and γd. This is actually another prediction from our model, the idea that species which rely more on external cues for searching will exhibit more 'asynchronous' search patterns than those lacking this ability or those adapted to more uncertain/variable conditions.

Let us note that we considered the option to include in our work an extra Section discussing in a more systematic way the possible effects that environmental conditions (not just cognitive cues, but also predation pressure, resources availability, etc) may have on our results. However, we finally discarded that option as such a discussion would be too speculative if based only on the present results. A further examination of the model in variable contexts would be necessary for that, so we decided to leave that idea for a future work.

Changes made: We have now introduced a brief discussion on this point in the Discussion Section, as a part of the future possible natural extensions of our present work.

l.113. What does "significant statistics" mean in that context?

Changes made: We have rephrased this sentence in order to clarify this point.

l.115. It is unclear at this point what the computational model is going to be used for. It would be useful to have a small section at the end of the introduction that lays out the main steps of the analyses, and explains their logic. The section l.174-177 does this but only after methods, could the same information be given verbally ?

Changes made: We have partially introduced the 'logic' requested by the reviewer at the end of the Introduction Section and in the beginning of Section IIB. We did that way to avoid discussing details within the Methods Section, as this point would remain possibly unnoticed there.

l.123. I do not understand the notions of "persistence" and "diffusive scaling" in this context, and a quick google search did not help. Please give more explanations. Alternatively, it seems to me that maybe these sentences could be removed entirely, since it is enough to know that food items are place far away from the nest.

Certainly, we used here terms that are very familiar in the field of statistical physics and movement ecology, but can be obscure to a more general audience. In short, "persistence" refers to the capacity of an individual to maintain its direction of motion for some time (this can be either measured as a characteristic time or as a characteristic distance covered without changing direction significantly). On the other side, 'diffusive scaling' is the term used to englobe all movement patterns in which the Mean Square Displacement scales linearly with time at long timescales (as is the case of Brownian motion, persistent random walks, and many other random models typically used to describe animal movement).

Changes made: We have rephrased this sentence by including a very short interpretation of these terms, and have cited some basic references as an introductory account to the field for the general reader.

l.143. This sentence is grammatically incorrect. More importantly, neither organisms nor groups maximize their fitness. The fitness quantity that is likely to be maximized in an evolving population is the growth rate of gene lineages. Depending on the system, this quantity sometimes aligns with individual fitness (number of offspring) or group productivity, but this sentence is misleading as is.

The paper cited (ref 42) makes exactly that point, and it is more about which currency we can use as fitness proxies when studying a system, than about the true quantity that selection optimizes.

We thank the reviewer for such an insightful comment. We mostly agree with it, and we admit that sentence was probably naïve as it was written.

Changes made: We have now tried to be more precise on this point. Foraging strategies used by real individuals/groups should be expected to maximize their growth rates, and then the relevant question is how to find a proper fitness function that correlates best with such rates. l.153, 165. It would be useful to see the mathematical definition of averages over random trajectories and time. I have a hard time understanding exactly how quantities in brackets where calculated.

Changes made: We have now rephrased this sentence by including an explicit example to facilitate understanding on this point.

l.159 Processing time is not considered either. This could be especially important in species such as A. senilis, that can sometimes rely on large food items. Please discuss.

We are not completely sure what the reviewer means by 'processing' time. We understand she refers to the possibility that food items need to be processed somehow to be transported to the nest from their position. In such case, we were implicitly considering this as part of the 'gathering' time, but we agree we could be more explicit on this point. In any case, the model relies on the assumption that timescales of 'gathering' , 'processing', and any other involved in the foraging process will be relatively short compared to the search time.

Changes made: We have rephrased this sentence to include the reviewer's suggestion, now being more explicit about the assumption used in the model.

l.168. Since the energy balance increases with α_i, I found it confusing that this parameter is referred to as a cost. If nothing prevents it, please modify.

Changes made: We named α_i relative intake gain, and α_f relative foraging cost. More specifically: *"These parameters α_{i} and α_f then represent the relative \textit{intake} gain and \textit{foraging} cost".*

l.170. species

Changes made: We have corrected this typo

l.180-219. This section shows how observations of lab colonies have yielded important qualitative information, and how this information was used to guide the modeling work (l.227 as well). I think it would be worth stating this early in the main text. As is, I sometimes had a hard time understanding the links between the experimental and mathematical aspects of the article.

We thank the reviewer for this comment. Actually, trying to link experimental and computational parts of the work was also the main point of discussion we found during preparation of the manuscript, as the choice was not trivial.

Changes made: We have rephrased several parts of the Introduction, and have additionally introduced a new paragraph at the beginning of the Results Section as a reading guide to facilitate linking and understanding of the different parts of the manuscript.

FIG2. To give a better account of statistical power, error bars should give the confidence intervals associated to the computed averages, not standard deviations.

Changes made: We have revised Fig.2 so that the error bars correspond to the 95% confidence intervals of the computed averages, replacing the previously used standard deviations,

L.221 It remains unclear to me why simulations where used only to determine Tfp and Tc, before plugging these values in eq. 1-5 to determine energy balance. Surely, energy balance could be determined from simulation directly? This would yield values less dependent on approximations, and could even be used to include more complex processes. Surely I am missing something here, please briefly explain.

This is certainly an interesting possibility we did not consider previousy. As some of the terms on the energy balance depend nonlinearly on Tfp and/or Tc, then computing mean times and plugging this into the model is not exactly equivalent to computing the mean energies (though the differences should not be high, in principle). To check this, we generated again Fig. 3 but now using this alternative method suggested by the referee. The results are presented in the plots below, where the plot in the left corresponds to the mean energy balance computed as suggested by the referee, while the plot in the right corresponds to the case used in the manuscript, using in both cases the same parameter values (α_i, α_f, v, Δt). We observe that, despite some small differences in the energy values, the conclusions obtained are exactly the same: (i) Energy balance tends to maximize for low gamma_d and large \gamma_b. (ii) For a fixed workforce (white diagonal lines, showing cases where 5% and 20% of the colony can be potentially allocate to foraging, respectively) there is an intermediate point with the maximum positive energy balance (iii) Changing the workforce translates into a shift of that maximum, which is much noticeable in the \gamma_b dimension, while changes in \gamma_d are much smaller.

Changes made: Since there is no clear argument to favour one version over the other (it simply depends on how the energy average has to be understood), we have decided finally to keep the original version in the manuscript, but rephrasing the sentence mentioned by the referee to include the current information discussed here.

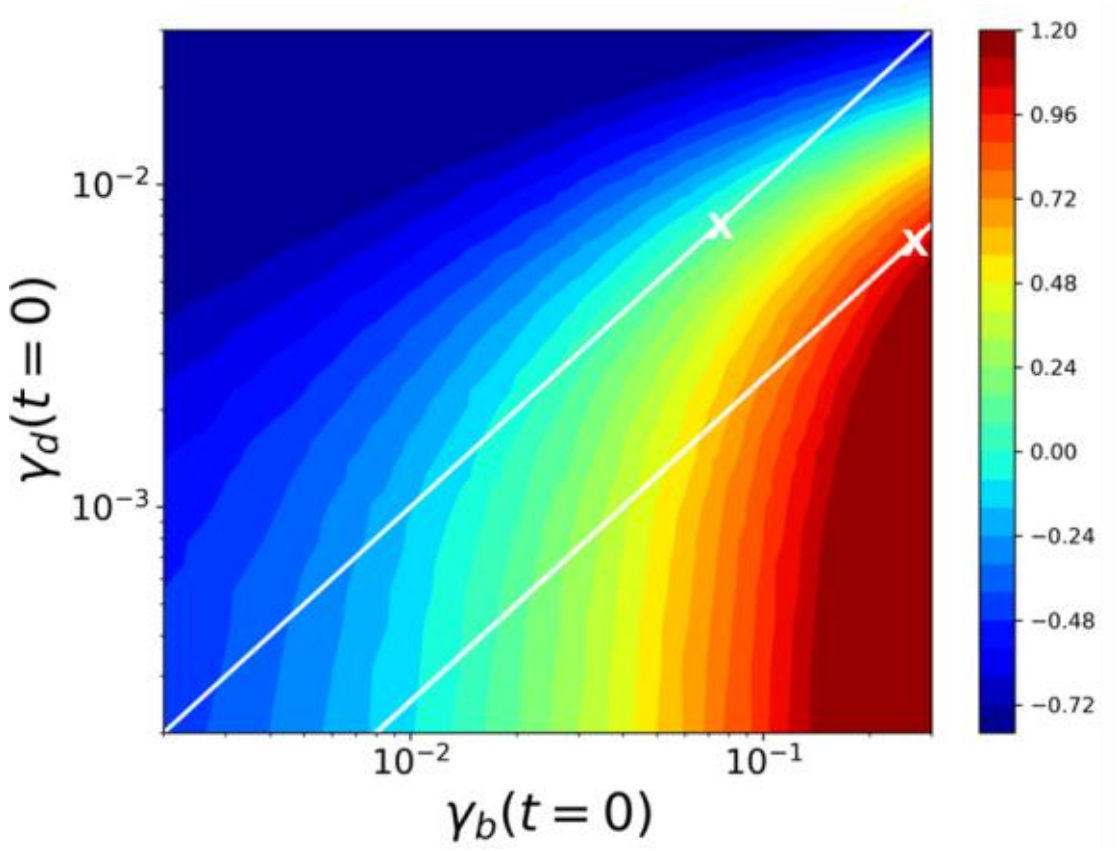

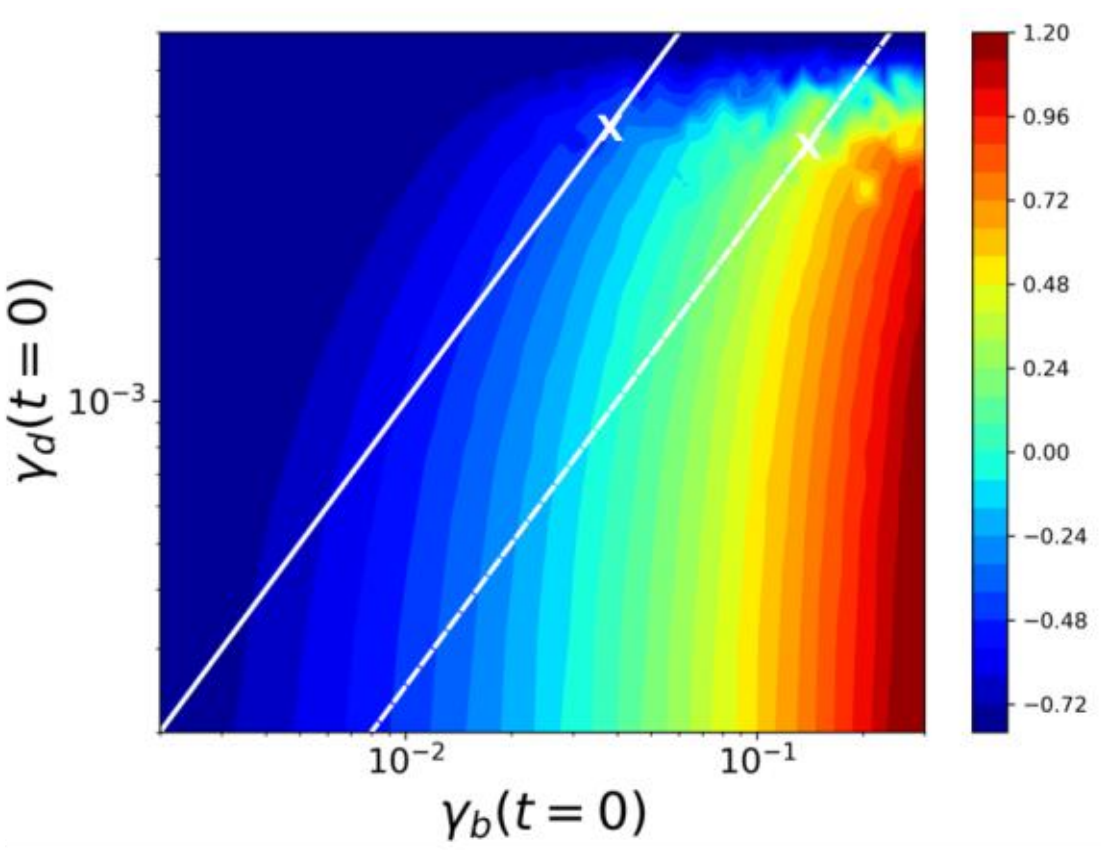

l.229. What is the motivation behind a two-fold change in parameters. Why not a larger or smaller change?

We chose this just on the basis of typical variations we observe in the experimental data (Fig.2). While we admit this is rather arbitrary, we have checked that three-fold changes or other choices would not yield any significant difference at a qualitative level.

Changes made: We have now included a sentence in this Section explicitly saying that we have checked this arbitrary choice does not significantly modify the discussion and conclusions reported.

FIG3. To be honest, it is hard for me to detect differences between the different panels of this figure.

Is it certain that a different scaling/cropping would not help making these differences more evident?

Changes made: We have tried rescaling the figure. However, since the differences are still relatively small we have additionally added a grid in the plots as a visual reference. Combining both things we think that now the readability of the figure has improved considerably.

l.249. I do not see how assuming that the colony has access to a maximum workforce account for predation. Please explain.

Changes made: We have changed this paragraph: "*However, our simulations do not explicitly incorporate the costs or risks of predation, nor other detrimental effects that can drop, and therefore limit the number of active ants involved in long-time massive collective deployments. We can indirectly account for these dynamic effects on the workforce load by assuming that the colony has access to a limited (maximum) workforce (N_w).*"

for this other one:

"While predation, environmental disturbances, and other detrimental factors are not explicitly modelled, their net effect of reducing the number of active foragers can be implicitly captured by the maximum workforce parameter (N_w), which represents an upper bound on the number of ants available for sustained collective foraging."

l.251. typo: γb)and(γd).

Changes made: We have corrected the typo

l.274. It also highlights that the model is missing some aspects of colonial life history. What would be the mechanisms that let small colonies persist and grow in natural populations, and that are not accounted for here?

Changes made: We have added a bit of a discussion based on this ideas/limitation at the end of the results section:

"The model omits several colonial life history traits that are likely to modulate foraging dynamics in natural populations, including worker turnover rate and experience, colony lifespan, foraging range, and tolerance to environmental stress. These factors may help explain how colonies persist and grow despite apparent limiting factors in collective

foraging, like the relative numbers of active workers compared to colony size (N_w/N_col). The observation that small colonies tend to commit a larger proportion of their workforce to active foraging suggests a compensatory strategy, but the mechanisms determining the minimal number of active workers required, whether driven by nutritional stress, worker experience, or demographic constraints, remain unaccounted for in the current framework."

l.309. What testable hypotheses emerge here for instance?

Changes made: Based on these comments, we have improved our initial paragraph of the Discussion section:

While there is substantial evidence that eusocial colonies can adjust their behaviour in response to environmental perturbations, most documented cases focus on traits such as nest-site selection or resource exploitation. The specific modulation of search patterns through workforce load variation has received comparatively little attention. Our model addresses this gap but does not capture the full complexity of factors that may influence search efficiency in natural colonies, including behavioural plasticity, worker experience, or key life history traits.

Nevertheless, a central contribution of this framework is to generate concrete, testable predictions about how the balance between active, high-metabolic-cost foragers and inactive, low-metabolic-cost workers shapes collective search efficiency. By making this trade-off explicit, the model provides a comparative baseline against which the contribution of other biological factors can be evaluated, and opens the way for empirical tests of how colonies of different sizes regulate their active foraging fraction under natural conditions.

l.312. What are high- and low-metabolism workers? do you mean foraging vs nest-bound?

Changes made: We have explained this better: *"In our framework, we capture this in its simplest form by distinguishing two metabolic states: active foragers, engaged in costly search behaviour outside the nest, and the rest, whether occupied with nest-bound tasks or waiting in the arena to be recruited."*

l.322 typo ","

Changes made: We have corrected the typo

l.324-328. To me, these two last sentences contradict each other. If larger colonies are on a tight budget, they should be less able to maintain a stationary reserve workforce. What am I missing here?

Our wording was clearly confusing, apologies. But the reviewer rationale is correct: As the colony size increases, there is more need of active foragers, which in turn are likely more energetically demanding , so that , to maintain a positive and buffered energetic balance one option would be to dynamically pool from alternative workforces in specific moments (a stationary reserve workforce) rather than have a fixed large proportion of active foragers (a fixed workforce load on foraging).

The problem of understanding comes from a confusing use of the word "workforce" in the previous text. We used "workforce loads" to refer to the active foragers and "stationary reserve workforce" to refer to those ants that are not foraging and are seemingly passive, waiting to be recruited for a foraging task.

Changes made: We have modified the previous paragraph to make this rationale more clear:

In other words, environments that allow colonies to thrive may simultaneously impose stricter limits on the proportion of workers that can be continuously engaged in active foraging as colony size increases. While small colonies can remain viable by deploying only a modest number of active searchers, larger colonies face a greater energetic burden per forager, making a high fixed proportion of active foragers increasingly costly to sustain. This scaling effect suggests that, for large colonies, maintaining a reserve workforce, a pool of inactive workers that can be facultatively recruited into foraging tasks when needed, becomes an advantageous, perhaps even necessary, evolutionary strategy. Rather than committing a large fixed fraction of workers to continuous foraging, dynamic recruitment from this reserve allows larger colonies to buffer energetic costs while preserving responsiveness to environmental demand.

l.341. Do you mean αi?

Yes, there were a couple of sentences where this typo appeared.

Changes maed: We have checked the whole document to address this and other problems with notation.

l.354. What is the "opposite pattern"?

We are making reference to the fact that in other species foraging patterns in general depend on group size. In the reference paper they explain how patch giving-up time variates for solo and grouped rodents.

Changes made: We have slightly modified the sentence to make this more clear:

In contrast, small groups of non‑eusocial species (e.g., rodents) often exhibit group size-sensitive foraging patterns~\cite{carthey2015}, highlighting the distinct collective dynamics that emerge in eusocial systems.l.358. Group selection is at best an unfortunate simplification, with no serious empirical or theoretical ground. The existence of coherent group-level behaviour (thus favouring every individual within it) can be explained fully by gene-level selection. Please rephrase.

Changes made: We just took out the word "group" from the sentence.

"If we assume that collective cognitive programs are themselves subject to selection, a view that is increasingly supported in the ecological literature \cite{torney2011,Bartumeus2009,evans2014,hintz2018,gauzens2021,ledru2023}, then our results offer a natural explanation for....."

Here, we wanted to highlight only that the "collective phenotype" can be subject to selection, without needing to enter at what level this process is taking place.

Reviewer #3:

1. Lines 129: In general, I think this algorithm could be clearer. For instance, at step iib) you say that the gaussian displacement has standard deviation v/ \Delta t, which has incorrect units if the random variable drawn being drawn is a displacement.

We thank the reviewer for detecting this typo; obviously we meant 'standard deviation v \Delta t'.

Changes made: We have rephrased the steps in the algorithm to make them clearer.

2. Line 144: in the phrase "biologically relevant magnitude", I think "magnitude" is not the correct word. I would suggest "biologically relevant metric" instead.

We thank the reviewer for this suggestion. Actually, Reviewer 2 made a few additional remarks about this sentence, so in the present version we have rewritten it completely.

Changes made: Following Reviewer 2, we now state there that foraging strategies used by real individuals/groups should be expected to maximize their growth rates, and then the relevant question is how to find a proper fitness function that correlates best with such rates.

3. Line 152: Manuscript claims that "Given our definition for the time required to find a food item, T_fp, the average number of items that can be obtained by the colony in a single day is then T_f /⟨T_fp⟩".

I don't understand how this follows except in the highly asynchronous limit. T_fp is defined as a first passage time, the time for the first trajectory to find the resource. Surely, if there are multiple simultaneous trajectories, then on average the time to find the resources the n-th time will be lower?

Perhaps, I am misunderstanding the way you are averaging over random trajectories?

Regardless, all of these operations should be defined explicitly.

We appreciate this comment, which let us see that probably this part of the model was not clear enough. First of all, T_fp represents the first-passage time of the overall search process (which may include multiple trajectories). Once that first-passage occurs (so food is detected by an individual), we are implicitly assuming that eusocial colonies have the capacity to communicate that information fast enough, so immediately processes of recruitment, communication between mates, etc, become activated to exploit the resource. So, there is no need to wait the n-th hitting time for the process to be complete. While this is obviously an approximation, our experimental datasets confirm that it is quite reasonable, as in the conditions we used first-passage takes typically 15-40 minutes, while recruitment seems to happen always in less than 5 minutes.

Changes made: We have included several sentences in Section IIC to make this approximation explicit and clarify the interpretation of Eq. (1).

4. In the captions for figures 3, 8, 9, the variable D=1 is used, but I don't think this is defined in the model section.

We apologize for this typo, which is due to a change in notation we introduced in the last version of our manuscript. D=1 should be correspondingly replaced by v=1.

Changes made: We have corrected this typo, and have checked that notation is unified now throughout all the text including Figure legends and captions.

5. The model text says N_a(t) reaches the stationary value \frac{\gamma_b}{\gamma_d} (line 132). I would expect to find that more active searchers reduces the time to find resources, and thus, is lowest when \gamma_b is large and \gamma_d is small.

However, what the authors have observed is roughly the opposite! The manuscript text reads "These plots reveal that the first-passage-time T_fp (Fig. 8) is lowest when \gamma_b is small and \gamma_d is large."

Why is this? I don't think the text addresses this.

We thank the reviewer for this comment. In connection to the comment 7 below, this is simply due to a typo in the labels of the axis, so \gamma_b and \gamma_d should read the contrary.

Changes made: We have corrected the typo in Figs. 8 and 9

6. In fig.3, fig. 8, and fig. 9, there are several smaller plots to the right that illustrate increasing/decreasing \gamma_b or \gamma_d through the simulation. These plots show almost identical results to the constant case and I don't think they add any additional insight. For simplicity I think they can be removed.

We think it is important to keep these plots as they show the effects of increasing/decreasing the two rates, so reflecting the trends that real colonies exhibit during the foraging process (Fig. 2). While it is true that differences between plots in the previous version of the manuscript were not large, this can be easily solved just through a different choice of parameter values and rescaling such plots.

Changes made: We have used a different scaling in the plots corresponding to non-constant rates \gamma_b or \gamma_c. .

7. The axes of fig.3 are flipped from fig.8 and fig.9, this is confusing.

As stated above, there was an unfortunate typo in Figs. 8 and 9 so that the labels appeared switched.

Changes made: We have corrected the typo, so now Figs 3, 8 and 9 share the same structure and compare easily.

8. In `README.txt` from the supplied code repository, it states that all averages were done over 1000 realizations, but I don't think this is mentioned in the text. Other important details of the simulation are also missing from the text, i.e. timestep.

Changes made: We have added the relevant technical details of the simulations in the main text, including the number of realizations used for averaging and the timestep employed, to facilitate reproducibility of the results.

9. On line 95, the authors claim that "additional datasets were obtained from a previous setup [41]", but the setup details in this manuscript appear to differ from the ones actually written in [41].

We would appreciate it if the reviewer could specify the discrepancies observed, as we have checked that the information appearing in Ref. [41] and the present manuscript are consistent. Maybe there is a misunderstanding about the arena size (we mentioned it was 2x2 meters in [41], while here we say it its 2x1). This is because in [41] we gave the whole size of the setup, which includes 2 different arenas. So, the correct arena size is 2x1.

10. On line 165, the authors claim that is approx. _t . This approximation should be justified somehow, it is not obvious to me.

The approximation presented in line 165 states comes from the fact that both N_a and T_fp are random variables (their value changes from one realization of the simulation, or each realization of the foraging process, to another). So that, <N_a T_fp>_t is the time-average of the product of two random variables. If N_a and T_fp are independent, then it follows that the time average of this product is the product of individual averages, <N_a T_fp>_t = <N_a>_t <T_fp> (the time average of T_fp is ill-defined as T_fp is itself a time, so an average over realizations must be used instead). But if these two variables are not independent (as is the case) then this identity is no longer true.

In consequence, the approximation used means we are assuming that correlations between N_a and T_fp are not too strong, so the condition of independent variables can be used.

Changes made: We have rephrased this sentence to make it explicit the assumption one is making when this approximation is used.

11. Various notation and spelling issues, i.e. \alpha_i written as \alpha_1, T_c is written as T_C sometimes, line 101 says "recourses" when it should say "resources" etc, "AKNOWDLEGMENTS" heading is misspelled.

Please proofread your manuscript.

Changes made: We have carefully revised notation and writing of the manuscript to detect and correct these mistakes

12. Incomplete citation issues: i.e.

[12] Unspecified authors, Unspecified Journal (2021).

[32] Unspecified authors, Journal of Ethology 31, 61 (2013).

Again please proofread your manuscript.

Changes made: We have sistematically revised the bibliography to detect and correct these mistakes

13. In figure 3 caption, the constraints the authors make are actually constraints on the stationary mean number of workers, as they constrain only the birth and death parameters of process. This should be made clear.

Changes made: We have tried to clarify this point both in the figure caption and in the main text.
