# Supplementary material for: Dynamic workforce modulation and foraging efficiency in Eusocial insect colonies
Source: PLoS Comput Biol. 2026 Oct 9;22(10):e1014258. doi: 10.1371/journal.pcbi.1014258 (PMC13667929; doi:10.1371/journal.pcbi.1014258)
Supplement: Supplementary file 5 [file pcbi.1014258.s007.pdf]

Reviewer #2:

Overall, I think the authors have provided a satisfactory revision. They have thoroughly addressed the issues raised by me and the two other reviewers. I found the revised version of the article much easier to read and understand. After they address the few minor comments given below, I believe the manuscript to be ready for publication.

All references show as question marks in the revised version, please proofread the pdf constructed by the editor (I am guessing the issue was introduced there) before sending the revision.

l.67. Here the required modification has not been implemented.

l.377-379. Based on this new sentence, I believe I have failed to get my point across completely. Maybe the best option at this point is to remove this sentence completely and make no statement about optimization. If a statement has to be made, just say that your are only interested in whatever optimizes colonial energetic balance, and neglect cases where natural selection leads to other outcomes.

l.421 ACKNOWDLEGMENTS This typo was raised by another reviewer already

Regarding the use of simulations to obtain energy balances directly: I believe there is a clear argument in favor of doing so! Simply put, it leads to more exact results (one less approximation). I understand that the authors do not want to change their methodology so late in the process (and I do not ask that they do), put please consider it for future projects.

We appreciate these additional comment, that have been useful to refine several details of the work.

Changes made: We have rephrased sentences in lines 67 and 377 following the reviewer advice, and we have additionally corrected the typo in line 421.

Regarding the final methodological comment, while it is true that we could save an approximation by doing as suggested by the reviewer, the method implemented in the text has the advantage that one can investigate the effect of the different terms contributing to the energy balance in a easier and more direct way. Actually, this has been quite useful through the design process leading to Figure 2. So, we rather prefer to keep this in its current form.
