# Supplementary material for: Dynamic workforce modulation and foraging efficiency in Eusocial insect colonies
Source: PLoS Comput Biol. 2026 Oct 9;22(10):e1014258. doi: 10.1371/journal.pcbi.1014258 (PMC13667929; doi:10.1371/journal.pcbi.1014258)
Supplement: Supplementary file 6 [file pcbi.1014258.s001.zip › S1 Text/S1_Text.pdf]

SUPPLEMENTARY INFORMATION: Dynamic Workforce Modulation and Foraging Efficiency in Eusocial Insect Colonies

Daniel Campos[1], Javier Cristín[1], Pol Fernández-López[2], Roger Lloret-Cabot[2], Meritxell Genovart[2], and Frederic Bartumeus[2,3,4]
[1]*Grup de Física Estadística, Departament de Física. Facultat de Ciències, Universitat Autònoma de Barcelona, 08193 Bellaterra (Barcelona), Spain.*
[2] *Centre d'Estudis Avançats de Blanes (CEAB-CSIC), Blanes, Girona, Spain*
[3] *ICREA, Institut Català de Recerca i Estudis Avançats, Barcelona, Spain.*
[4]*Centre de Recerca Ecològica i Aplicacions Forestals (CREAF), Cerdanyola del Vallès, Barcelona, Spain*
(Dated: September 24, 2026)

I. ADDITIONAL EXPERIMENTAL RESULTS

In this Appendix we complement the empirical results discussed in Section III to identify relevant questions related to their significance. In particular, we focus on how (i) the results obtained for the initial (search plus initial gathering) phase of foraging become modified as later phases are reached, and (ii) the implications and aspects related to the variability between colonies/experiments.

In Fig A we show the values of the rates γ_b and γ_d obtained for the whole duration of the experiments (up to 120 minutes), so including later phases of the foraging process (essentially the end of the gathering phase and the pos-gathering period). It corresponds to a situation where the colony is actively exploiting (or has already exploited) food resources after the search phase, and so the colony behavior is a combination of (i) additional exploration for further resources, and (ii) relaxation dynamics once the probability to find them is low enough. Consequently, colonies exhibit in general a decreasing activity during that period (though the intensity of this strongly depends on the particular colony and the food conditions). This reflects into a certain decrease in the value of γ_b (so less active trajectories emerge out of the nest), but surprisingly γ_d does not increase significantly, or even go on decreasing during the final part of the experiments (except in the case of colony B1). Then, this stage is characterized by long trajectories of a few (specialized, probably) foragers trying to reach further regions of the arena, while the recruitment of new workforces remains at low levels.

Fig B, finally, shows the dispersion of results for $N_a(t)$ that we observe between individual experiments. While variability is considerable, it is remarkable that the characteristic regions occupied by each colony show low levels of overlap, which confirms our conclusion above that intercolony variability is much more significant than differences due to different food conditions and/or interday fluctuations. This means that adaptation responses of the groups are not immediate within the timescales of our experiments. It remains then to be explored experimentally how fast the colonies would be able to modify such strategies and/or what the main drivers are that would activate such adaptation.

II. EFFECT OF WORKFORCE MODULATION ON SEARCH DYNAMICS

As stated in Section V, we computed random walk simulations of the search process with variable workforce to extract the values for the first-passage time T_{fp} (defined as the time required for any of the active foragers to find the resource) and the Collective Time Cost (T_C, defined as the sum of the durations of all search trajectories in the simulation up to time T_{fp}). These two are the only parameters in the energy balance that are computed numerically (the rest being chosen arbitrarily to facilitate visualization of our results).

By examining how $\langle T_{fp} \rangle$ and $\langle T_C \rangle$ depend on the rates γ_b and γ_d, we gain additional insight into how search dynamics affect the colony's energy balance. Figures C and D show the corresponding heatmaps for the same scenario illustrated in Fig 2 of the main text, but now for $\langle T_{fp} \rangle$ and $\langle T_C \rangle$, respectively. These plots reveal that the first-passage-time T_{fp} (Fig C) is lowest for γ_d small and γ_b large. As γ_d increases, achieving comparable search efficiency requires increasingly larger γ_b. Consequently, the balance between departure and return rates shapes the timescales over which food is found.

In some regions of parameter space, the search effort becomes insufficient to locate the resource, leading to a pronounced increase in times required to find the resource (so, a decline in search efficiency). The regime characterized by low γ_b and γ_d reflects the idea that slowly increasing the number of scouts in the arena and subsequently keeping them synchronized there allows colonies to reduce resource detection times. Compared with stationary rates, non-stationary

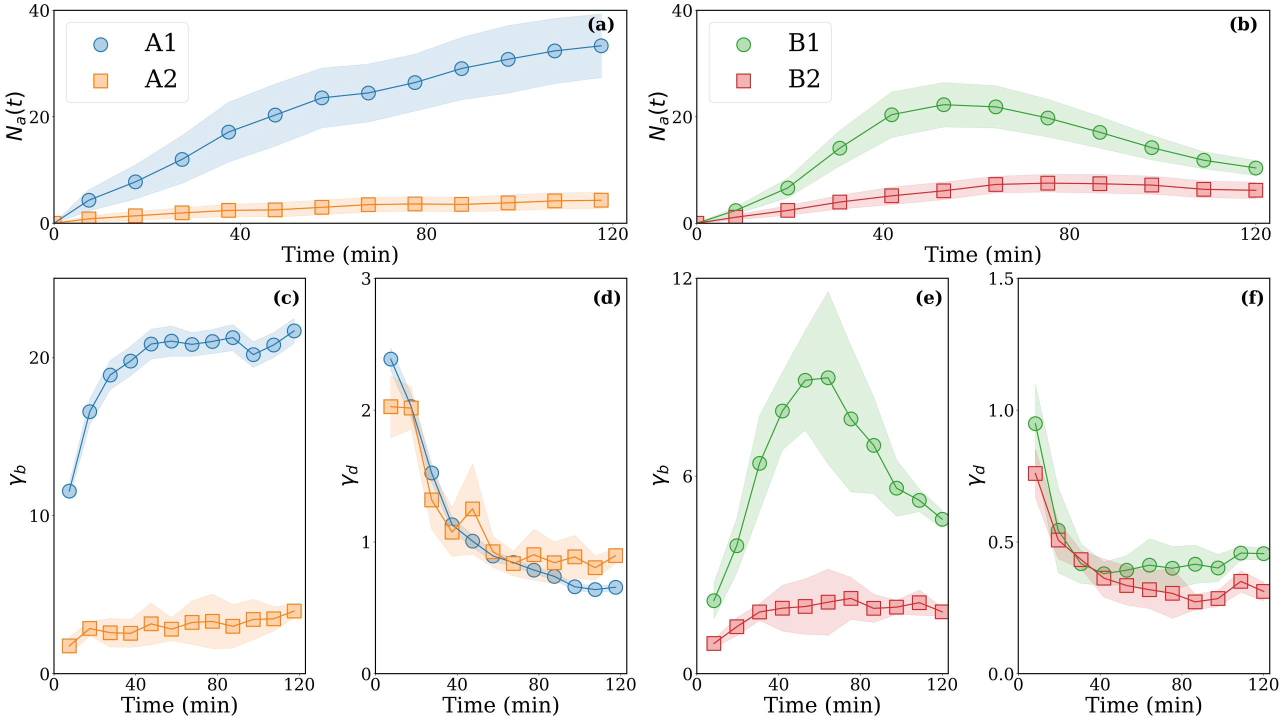

Fig A. Average number of ants $N_a(t)$ and rates γ_b and γ_d obtained from the experimental datasets for the four colonies considered, two for experiment A (left) and two for experiment B (right). Patterns are shown for the full duration of experiments (120 minutes) to be compared with Fig **??** showing the first 60 minutes of experiment. Error bars (shaded region) correspond to the 95% confidence interval of the mean, computed from all daily experiments carried out for each colony.

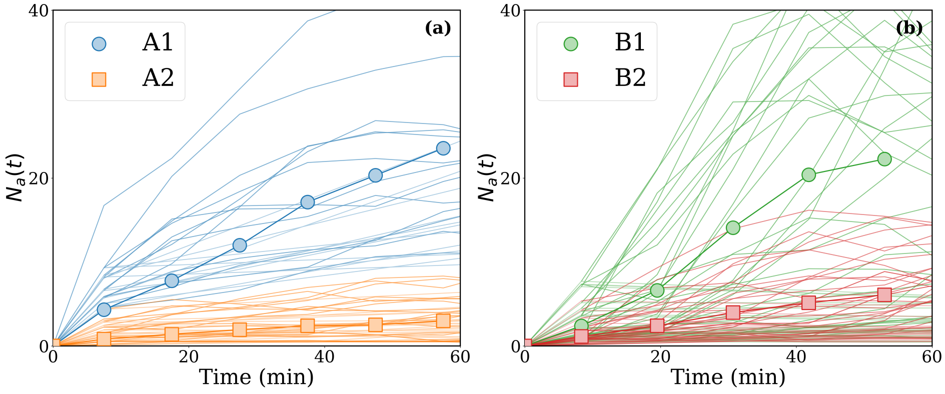

Fig B. Average number of ants $N_a(t)$ obtained from the experimental datasets for the four colonies considered, two for experiment A (left) and two for experiment B (right). The light lines correspond to data of the daily experiments carried out for each colony.

departure and arrival rates (right panels in Fig C) noticeably affect the first-passage-time efficiency. Increasing (decreasing) γ_b (γ_d) over time slightly improves search times; however, the dominant effect is that increasing return rates (γ_d) while decreasing departure rates (γ_b) leads to an overall reduction in first-passage-time efficiency.

Opposite to the results for the first-passage times, the collective search time T_C is minimized (i.e., efficiency is maximized) in the regime of low (γ_b) and low (γ_d) (Fig D). Excessively large departure rates would impose an unnecessary workforce cost, whereas excessively large return rates would prevent collective search trajectories from remaining in the arena long enough to improve resource detection. Notably, decreasing γ_b and γ_d over time (right panels in Fig D) expands the region of low T_C. In other words, the cost of collective search is reduced (i.e. search efficiency improves) when the system begins with a rapid turnover of trajectories that subsequently slows down, ensuring that workers in the nest are recruited only after previous trajectories have had sufficient time to travel far

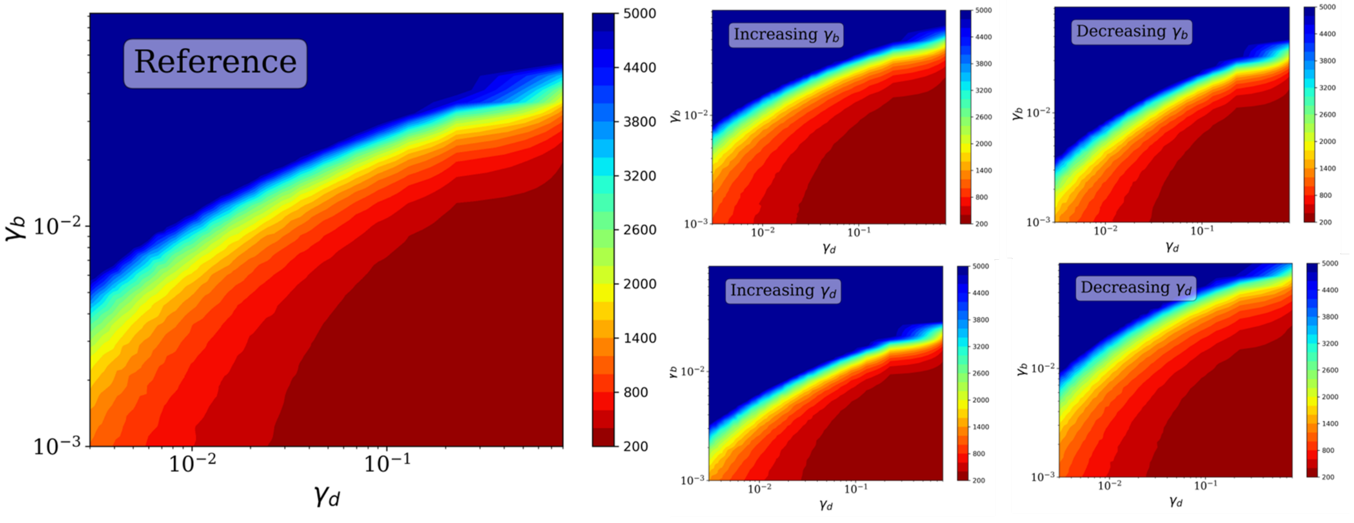

Fig C. Heatmaps obtained from random-walk simulations for the the mean first-passage time, $\langle T_{fp} \rangle$ through a target located at distance $d_r = 10$ (with $v = 1$ and $d_d = 1$) as a function of rates γ_b, γ_d. (Left): reference case where γ_b, γ_d are kept constant during every simulation. (Right): Cases with either $\gamma_b(t)$ or $\gamma_d(t)$ increasing or decreasing by a factor of two during the search. Such an increase or decrease is linear with time and terminates after a characteristic time $d_r^2/2D$, so after that time γ_b and γ_d will remain constant for the rest of the simulation.

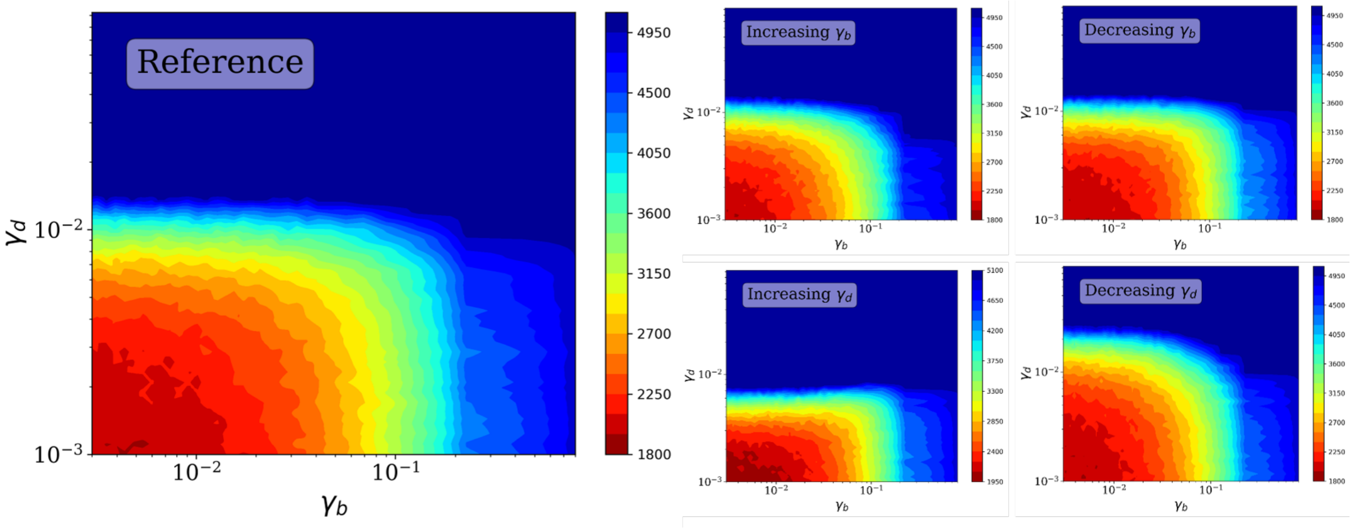

Fig D. Heatmaps obtained from random-walk simulations for the collective search time, $\langle T_C \rangle$, through a target located at distance $d_r = 10$ (with $v = 1$ and $d_d = 1$) as a function of rates γ_b, γ_d. (Left): reference case where γ_b, γ_d are kept constant during every simulation. (Right): Cases with either $\gamma_b(t)$ or $\gamma_d(t)$ increasing or decreasing by a factor of two during the search. Such an increase or decrease is linear with time and terminates after a characteristic time $d_r^2/2D$, so after that time γ_b and γ_d will remain constant for the rest of the simulation.

from the nest.

The energy balance described in the main text then reflects an interplay between these two components: the first-passage time, which favours resource-rich (or easy resource deteciton) and synchronous search, and the collective cost, which favours more resource-limited (or hard-to-find resources) asynchronous search strategies. This is further modulated by the basal energy consumption, which scales with colony size. Consequently, the energetic availability of the colony and the optimal energy gain ΔE_X studied throughout the article emerge from a nontrivial interaction among all these elements.
