# Supplementary material for: Dynamic workforce modulation and foraging efficiency in Eusocial insect colonies
Source: PLoS Comput Biol. 2026 Oct 9;22(10):e1014258. doi: 10.1371/journal.pcbi.1014258 (PMC13667929; doi:10.1371/journal.pcbi.1014258)
Supplement: Peer review history

# Peer review: Decision Letter 0

**DOI:** 10.1371/journal.pcbi.1014258.r001

**Authors:** Philipp Altrock, Tobias Bollenbach

26 May 2026

Dynamic Workforce Modulation and Foraging Efficiency in Eusocial Insect Colonies

PLOS Computational Biology

Dear Dr. Campos,

Thank you for submitting your manuscript to PLOS Computational Biology. After careful consideration, we feel that it has merit but does not fully meet PLOS Computational Biology's publication criteria as it currently stands. Therefore, we invite you to submit a revised version of the manuscript that addresses the points raised during the review process.

Please submit your revised manuscript by Jul 26 2026 11:59PM. If you will need more time than this to complete your revisions, please reply to this message or contact the journal office at ploscompbiol@plos.org. When you're ready to submit your revision, log on to https://www.editorialmanager.com/pcompbiol/ and select the 'Submissions Needing Revision' folder to locate your manuscript file.

Please include the following items when submitting your revised manuscript:

* A letter that responds to each point raised by the editor and reviewer(s). You should upload this letter as a separate file labeled 'Response to Reviewers'. This file does not need to include responses to formatting updates and technical items listed in the 'Journal Requirements' section below.

* A marked-up copy of your manuscript that highlights changes made to the original version. You should upload this as a separate file labeled 'Revised Manuscript with Track Changes'.

* An unmarked version of your revised paper without tracked changes. You should upload this as a separate file labeled 'Manuscript'.

As the corresponding author, your ORCID iD is verified in the submission system and will appear in the published article. PLOS supports the use of ORCID, and we encourage all coauthors to register for an ORCID iD and use it as well. Please encourage your coauthors to verify their ORCID iD within the submission system before final acceptance, as unverified ORCID iDs will not appear in the published article. Only the individual author can complete the verification step; PLOS staff cannot verify ORCID iDs on behalf of authors.

We look forward to receiving your revised manuscript.

Kind regards,

Philipp Martin Altrock, Ph.D.

Academic Editor

PLOS Computational Biology

Tobias Bollenbach

Section Editor

PLOS Computational Biology

**Journal Requirements:**

If the reviewer comments include a recommendation to cite specific previously published works, please review and evaluate these publications to determine whether they are relevant and should be cited. There is no requirement to cite these works unless the editor has indicated otherwise.

1) We ask that a manuscript source file is provided at Revision. Please upload your manuscript file as a .doc, .docx, .rtf or .tex. If you are providing a .tex file, please upload it under the item type u2018LaTeX Source Fileu2019 and leave your .pdf version as the item type u2018Manuscriptu2019.

2) Please provide an Author Summary. This should appear in your manuscript between the Abstract (if applicable) and the Introduction, and should be 150-200 words long. The aim should be to make your findings accessible to a wide audience that includes both scientists and non-scientists. Sample summaries can be found on our website under Submission Guidelines:

https://journals.plos.org/ploscompbiol/s/submission-guidelines#loc-parts-of-a-submission

3) Your manuscript's sections are not in the correct order.  Please amend to the following order: Abstract, Introduction, Results, Discussion, and Methods

4) Please upload all main figures as separate Figure files in .tif or .eps format. For more information about how to convert and format your figure files please see our guidelines:

https://journals.plos.org/ploscompbiol/s/figures

5) Some material included in your submission may be copyrighted. According to PLOS’s copyright policy, authors who use figures or other material (e.g., graphics, clipart, maps) from another author or copyright holder must demonstrate or obtain permission to publish this material under the Creative Commons Attribution 4.0 International (CC BY 4.0) License used by PLOS journals. Please closely review the details of PLOS’s copyright requirements here: PLOS Licenses and Copyright. If you need to request permissions from a copyright holder, you may use PLOS's Copyright Content Permission form.

Please respond directly to this email and provide any known details concerning your material's license terms and permissions required for reuse, even if you have not yet obtained copyright permissions or are unsure of your material's copyright compatibility. Once you have responded and addressed all other outstanding technical requirements, you may resubmit your manuscript within Editorial Manager.

Potential Copyright Issues:

i) Please confirm (a) that you are the photographer of 1, or (b) provide written permission from the photographer to publish the photo(s) under our CC BY 4.0 license.

6) Please amend your detailed Financial Disclosure statement. This is published with the article. It must therefore be completed in full sentences and contain the exact wording you wish to be published.

1) State what role the funders took in the study. If the funders had no role in your study, please state: "The funders had no role in study design, data collection and analysis, decision to publish, or preparation of the manuscript."

2) If any authors received a salary from any of your funders, please state which authors and which funders..

If you did not receive any funding for this study, please simply state: u201cThe authors received no specific funding for this work.u201d

**Reviewers' comments:**

Reviewer's Responses to Questions

**Comments to the Authors:**

**Please note here if the review is uploaded as an attachment.**

Reviewer #1: see attached file

Reviewer #2: In this article the authors explore the links between high-level colonial exploration parameters, the distribution of resources in the environment, and colonial productivity. With simple and overall well-explained methodology, combining modeling with experimental work, they highlight some of the core trade-offs that constraint foraging in eusocial groups. The theoretical approach is sound, but should be refined in the future to account for important aspects of colonial life history, such as variance in individual behaviour, predation, resource handling, or individual interactions during foraging.

The main limitation of the current manuscript, in my opinion, lies in the lack of higher-level discussion of the derived results. Too little effort is put in discussing the limitations of the model and experimental set-up, which may participate in heavily biasing the results. A good example is the result according to which large colonies have a harder time keeping their energy balance positive. This somewhat contrasts with common knowledge about ant colonies, which often produce large surplus only at larger sizes (due to better competitivity, access to different resources, synergy during foraging…). A better characterization of the model’s limits, in the face of the complexity and diversity of eusocial systems, is required for a better integration of the results.

More specific comments in the attached file.

Reviewer #3: 1. Lines 129: In general, I think this algorithm could be clearer. For instance, at step iib) you say that the gaussian displacement has standard deviation v/ \Delta t, which has incorrect units if the random variable drawn being drawn is a displacement.

2. Line 144: in the phrase "biologically relevant magnitude", I think "magnitude" is not the correct word. I would suggest "biologically relevant metric" instead.

3. Line 152: Manuscript claims that "Given our definition for the time required to find a food item, T_fp, the average number of items that can be obtained by the colony in a single day is then T_f /〈T_fp〉".

I don't understand how this follows except in the highly asynchronous limit. T_fp is defined as a first passage time, the time for the first trajectory to find the resource. Surely, if there are multiple simultaneous trajectories, then on average the time to find the resources the n-th time will be lower?

Perhaps, I am misunderstanding the way you are averaging over random trajectories?

Regardless, all of these operations should be defined explicitly.

4. In the captions for figures 3, 8, 9, the variable D=1 is used, but I don't think this is defined in the model section.

5. The model text says N_a(t) reaches the stationary value \frac{\gamma_b}{\gamma_d} (line 132).

I would expect to find that more active searchers reduces the time to find resources, and thus, <t_fp>is lowest when \gamma_b is large and \gamma_d is small.

However, what the authors have observed is roughly the opposite! The manuscript text reads "These plots reveal that the first-passage-time T_fp (Fig. 8) is lowest when \gamma_b is small and \gamma_d is large."

Why is this? I don't think the text addresses this.

6. In fig.3, fig. 8, and fig. 9, there are several smaller plots to the right that illustrate increasing/decreasing \gamma_b or \gamma_d through the simulation. These plots show almost identical results to the constant case and I don't think they add any additional insight. For simplicity I think they can be removed.

7. The axes of fig.3 are flipped from fig.8 and fig.9, this is confusing.

8. In `README.txt` from the supplied code repository, it states that all averages were done over 1000 realizations, but I don't think this is mentioned in the text. Other important details of the simulation are also missing from the text, i.e. timestep.

9. On line 95, the authors claim that "additional datasets were obtained from a previous setup [41]", but the setup details in this manuscript appear to differ from the ones actually written in [41].

10. On line 165, the authors claim that <t_c>is approx. <n_a>_t <t_fp>. This approximation should be justified somehow, it is not obvious to me.

11. Various notation and spelling issues, i.e. \alpha_i written as \alpha_1, T_c is written as T_C sometimes, line 101 says "recourses" when it should say "resources" etc, "AKNOWDLEGMENTS" heading is misspelled.

Please proofread your manuscript.

12. Incomplete citation issues: i.e.

[12] Unspecified authors, Unspecified Journal (2021).

[32] Unspecified authors, Journal of Ethology 31, 61 (2013).

Again please proofread your manuscript.

13. In figure 3 caption, the constraints the authors make are actually constraints on the stationary mean number of workers, as they constrain only the birth and death parameters of process. This should be made clear.</t_fp></n_a></t_c></t_fp>

**********

**Have the authors made all data and (if applicable) computational code underlying the findings in their manuscript fully available?**

The PLOS Data policy requires authors to make all data and code underlying the findings described in their manuscript fully available without restriction, with rare exception (please refer to the Data Availability Statement in the manuscript PDF file). The data and code should be provided as part of the manuscript or its supporting information, or deposited to a public repository. For example, in addition to summary statistics, the data points behind means, medians and variance measures should be available. If there are restrictions on publicly sharing data or code —e.g. participant privacy or use of data from a third party—those must be specified.

Reviewer #1: Yes

Reviewer #2: Yes

Reviewer #3: **No:**  1. The code that implements the actual random walk simulations is not included.

There is also none of the plotting code.

There are 8 python files in the Codes.zip folder in the repository, and none of them have more than 42 lines of python code. It is impossible to check if the code used to generate these plots even matches the model described.

2. The README states that "The mean*.dat files contains the average results corresponding to 1000 realizations of each subset of r_b and r_d values". I assume that r_b and r_d should be \gamma_b and \gamma_d. However, the actual values in the .dat files do not seem to align with the ranges of \gamma_b and \gamma_d used in the manuscript.

These data files also don't specify any of the other parameters used.

3. There is no script that reproduces exactly the simulations done in the manuscript. The authors write that one must manually adjust these scripts "Depending on the specific dataset or experiment, users may need to adapt file names, input paths, and parameter settings (e.g., time windows, number of files, or grouping of experiments)." A list of software dependencies should also be included. Ideally in an `requirements.txt` format or similar.

I do not think this meets the reproducibility guidelines for this journal. I would expect to see a python or bash file which runs the simulations for the exactly the parameter ranges and plots published in the manuscript.

**********

PLOS authors have the option to publish the peer review history of their article (what does this mean?). If published, this will include your full peer review and any attached files.

If you choose “no”, your identity will remain anonymous but your review may still be made public.

**Do you want your identity to be public for this peer review?**  For information about this choice, including consent withdrawal, please see our Privacy Policy.

Reviewer #1: No

Reviewer #2: No

Reviewer #3: **Yes:**  Peter Carl Jentsch

[NOTE: If reviewer comments were submitted as an attachment file, they will be attached to this email and accessible via the submission site. Please log into your account, locate the manuscript record, and check for the action link "View Attachments". If this link does not appear, there are no attachment files.]

**Figure resubmission:**

After uploading your figures to PLOS’s NAAS tool - https://ngplosjournals.pagemajik.ai/artanalysis, NAAS will process the files provided and display the results in the "Uploaded Files" section of the page as the processing is complete. If the uploaded figures meet our requirements (or NAAS is able to fix the files to meet our requirements), the figure will be marked as "fixed" above. If NAAS is unable to fix the files, a red "failed" label will appear above. When NAAS has confirmed that the figure files meet our requirements, please download the file via the download option, and include these NAAS processed figure files when submitting your revised manuscript.

**Reproducibility:**

To enhance the reproducibility of your results, we recommend that authors of applicable studies deposit laboratory protocols in protocols.io, where a protocol can be assigned its own identifier (DOI) such that it can be cited independently in the future. Additionally, PLOS ONE offers an option to publish peer-reviewed clinical study protocols. Read more information on sharing protocols at https://plos.org/protocols?utm_medium=editorial-email&utm_source=authorletters&utm_campaign=protocols

Attachment Submitted filename: campos0526.pdf

Attachment Submitted filename: Plos_Campos_2026.pdf

# Author comment: Author response to Decision Letter 1

**DOI:** 10.1371/journal.pcbi.1014258.r002

1 Jul 2026

Attachment Submitted filename: PLOS_Response.pdf

# Peer review: Decision Letter 1

**DOI:** 10.1371/journal.pcbi.1014258.r003

**Authors:** Philipp Altrock, Tobias Bollenbach

10 Aug 2026

PCOMPBIOL-D-26-00932R1

Dynamic Workforce Modulation and Foraging Efficiency in Eusocial Insect Colonies

PLOS Computational Biology

Dear Dr. Campos,

Thank you for submitting your manuscript to PLOS Computational Biology. After careful consideration, we feel that it has merit but does not fully meet PLOS Computational Biology's publication criteria as it currently stands. Therefore, we invite you to submit a revised version of the manuscript that addresses the points raised during the review process.

Please submit your revised manuscript by Oct 10 2026 11:59PM. If you will need more time than this to complete your revisions, please reply to this message or contact the journal office at ploscompbiol@plos.org. When you're ready to submit your revision, log on to https://www.editorialmanager.com/pcompbiol/ and select the 'Submissions Needing Revision' folder to locate your manuscript file.

Please include the following items when submitting your revised manuscript:

* recompile citations and check all bibliography references carefully.

* If necessary, write a point-by-point response to the final reviewer remarks.

* If changes are more substantial, summarize them in a cover letter.

If you would like to make changes to your financial disclosure, competing interests statement, or data availability statement, please make these updates within the submission form at the time of resubmission. Guidelines for resubmitting your figure files are available below the reviewer comments at the end of this letter.

As the corresponding author, your ORCID iD is verified in the submission system and will appear in the published article. PLOS supports the use of ORCID, and we encourage all coauthors to register for an ORCID iD and use it as well. Please encourage your coauthors to verify their ORCID iD within the submission system before final acceptance, as unverified ORCID iDs will not appear in the published article. Only the individual author can complete the verification step; PLOS staff cannot verify ORCID iDs on behalf of authors.

We look forward to receiving your revised manuscript.

Kind regards,

Philipp Martin Altrock, Ph.D.

Academic Editor

PLOS Computational Biology

Tobias Bollenbach

Section Editor

PLOS Computational Biology

**Additional Editor Comments (if provided):**

Please recompile the citations and check all references carefully.

**Journal Requirements:**

If the reviewer comments include a recommendation to cite specific previously published works, please review and evaluate these publications to determine whether they are relevant and should be cited. There is no requirement to cite these works unless the editor has indicated otherwise.

1) We ask that a manuscript source file is provided at Revision. Please upload your manuscript file as a .doc, .docx, .rtf or .tex. If you are providing a .tex file, please upload it under the item type u2018LaTeX Source Fileu2019 and leave your .pdf version as the item type u2018Manuscriptu2019.

2) Please provide an Author Summary. This should appear in your manuscript between the Abstract (if applicable) and the Introduction, and should be 150-200 words long. The aim should be to make your findings accessible to a wide audience that includes both scientists and non-scientists. Sample summaries can be found on our website under Submission Guidelines:

https://journals.plos.org/ploscompbiol/s/submission-guidelines#loc-parts-of-a-submission

3) Please upload all main figures as separate Figure files in .tif or .eps format. For more information about how to convert and format your figure files please see our guidelines:

https://journals.plos.org/ploscompbiol/s/figures

4) We have noticed that you have uploaded Supporting Information files, but you have not included a list of legends. Please add a full list of legends for your Supporting Information files after the references list.

5) Please amend your detailed Financial Disclosure statement. This is published with the article. It must therefore be completed in full sentences and contain the exact wording you wish to be published.

i) State what role the funders took in the study. If the funders had no role in your study, please state: "The funders had no role in study design, data collection and analysis, decision to publish, or preparation of the manuscript."

ii) If any authors received a salary from any of your funders, please state which authors and which funders..

If you did not receive any funding for this study, please simply state: u201cThe authors received no specific funding for this work.u201d

**Reviewers' comments:**

Reviewer's Responses to Questions

**Comments to the Authors:**

**Please note here if the review is uploaded as an attachment.**

Reviewer #2: Overall, I think the authors have provided a satisfactory revision. They have thoroughly addressed the issues raised by me and the two other reviewers. I found the revised version of the article much easier to read and understand. After they address the few minor comments given below, I believe the manuscript to be ready for publication.

All references show as question marks in the revised version, please proofread the pdf constructed by the editor (I am guessing the issue was introduced there) before sending the revision.

l.67. Here the required modification has not been implemented.

l.377-379. Based on this new sentence, I believe I have failed to get my point across completely. Maybe the best option at this point is to remove this sentence completely and make no statement about optimization. If a statement has to be made, just say that your are only interested in whatever optimizes colonial energetic balance, and neglect cases where natural selection leads to other outcomes.

l.421 ACKNOWDLEGMENTS This typo was raised by another reviewer already

Regarding the use of simulations to obtain energy balances directly: I believe there is a clear argument in favor of doing so! Simply put, it leads to more exact results (one less approximation). I understand that the authors do not want to change their methodology so late in the process (and I do not ask that they do), put please consider it for future projects.

Reviewer #3: My concerns have mostly been addressed, thank you.

**********

**Have the authors made all data and (if applicable) computational code underlying the findings in their manuscript fully available?**

The PLOS Data policy requires authors to make all data and code underlying the findings described in their manuscript fully available without restriction, with rare exception (please refer to the Data Availability Statement in the manuscript PDF file). The data and code should be provided as part of the manuscript or its supporting information, or deposited to a public repository. For example, in addition to summary statistics, the data points behind means, medians and variance measures should be available. If there are restrictions on publicly sharing data or code —e.g. participant privacy or use of data from a third party—those must be specified.

Reviewer #2: Yes

Reviewer #3: Yes

**********

PLOS authors have the option to publish the peer review history of their article (what does this mean?). If published, this will include your full peer review and any attached files.

If you choose “no”, your identity will remain anonymous but your review may still be made public.

**Do you want your identity to be public for this peer review?**  For information about this choice, including consent withdrawal, please see our Privacy Policy.

Reviewer #2: No

Reviewer #3: No

[NOTE: If reviewer comments were submitted as an attachment file, they will be attached to this email and accessible via the submission site. Please log into your account, locate the manuscript record, and check for the action link "View Attachments". If this link does not appear, there are no attachment files.]

**Figure resubmission:**

**Reproducibility:**

To enhance the reproducibility of your results, we recommend that authors of applicable studies deposit laboratory protocols in protocols.io, where a protocol can be assigned its own identifier (DOI) such that it can be cited independently in the future. Additionally, PLOS ONE offers an option to publish peer-reviewed clinical study protocols. Read more information on sharing protocols at https://plos.org/protocols?utm_medium=editorial-email&utm_source=authorletters&utm_campaign=protocols

# Author comment: Author response to Decision Letter 2

**DOI:** 10.1371/journal.pcbi.1014258.r004

19 Aug 2026

Attachment Submitted filename: PLOS_Response 2.pdf

# Editor report: Decision Letter 2

**DOI:** 10.1371/journal.pcbi.1014258.r005

**Authors:** Philipp Altrock, Tobias Bollenbach

21 Aug 2026

PCOMPBIOL-D-26-00932R2

Dynamic Workforce Modulation and Foraging Efficiency in Eusocial Insect Colonies

PLOS Computational Biology

Dear Dr. Campos,

Thank you for submitting your manuscript to PLOS Computational Biology.

The amnuscript is now acceptable by the reviwers, but please upload a pdf that has been correctly compiled so that all references are shown in the text. Only then can final editorial review occur.

Please submit your revised manuscript by Oct 21 2026 11:59PM. When you're ready to submit your revision, log on to https://www.editorialmanager.com/pcompbiol/ and select the 'Submissions Needing Revision' folder to locate your manuscript file.

We look forward to receiving your correctly compiled manuscript.

Kind regards,

Philipp Martin Altrock, Ph.D.

Academic Editor

PLOS Computational Biology

Tobias Bollenbach

Section Editor

PLOS Computational Biology

**Additional Editor Comments (if provided):**

**Journal Requirements:**

If the reviewer comments include a recommendation to cite specific previously published works, please review and evaluate these publications to determine whether they are relevant and should be cited. There is no requirement to cite these works unless the editor has indicated otherwise.

1) Please upload all main figures as separate Figure files in .tif or .eps format. For more information about how to convert and format your figure files please see our guidelines:

https://journals.plos.org/ploscompbiol/s/figures

2) We have noticed that you have uploaded Supporting Information files, but you have not included a list of legends. Please add a full list of legends for your Supporting Information files after the references list.

3) Thank you for stating the Data availability statement. However, we were unable to access the link (https://doi.org/10.34810/DATA3541)using the information provided. Please review and update the Data Availability Statement to ensure that the underlying data are publicly accessible and that all accession numbers, links, or repository details are correct and functional.

**Reviewers' comments:**

[NOTE: If reviewer comments were submitted as an attachment file, they will be attached to this email and accessible via the submission site. Please log into your account, locate the manuscript record, and check for the action link "View Attachments". If this link does not appear, there are no attachment files.]

**Figure resubmission:**

**Reproducibility:**

To enhance the reproducibility of your results, we recommend that authors of applicable studies deposit laboratory protocols in protocols.io, where a protocol can be assigned its own identifier (DOI) such that it can be cited independently in the future. Additionally, PLOS ONE offers an option to publish peer-reviewed clinical study protocols. Read more information on sharing protocols at https://plos.org/protocols?utm_medium=editorial-email&utm_source=authorletters&utm_campaign=protocols

# Author comment: Author response to Decision Letter 3

**DOI:** 10.1371/journal.pcbi.1014258.r006

26 Aug 2026

Attachment Submitted filename: PLOS_Response_2_auresp_3.pdf

# Editor report: Decision Letter 3

**DOI:** 10.1371/journal.pcbi.1014258.r007

**Authors:** Philipp Altrock, Tobias Bollenbach

3 Sep 2026

Dear Dr. Campos,

We are pleased to inform you that your manuscript 'Dynamic Workforce Modulation and Foraging Efficiency in Eusocial Insect Colonies' has been provisionally accepted for publication in PLOS Computational Biology.

Before your manuscript can be formally accepted you will need to complete some formatting changes, which you will receive in a follow up email. A member of our team will be in touch with a set of requests.

Please note that your manuscript will not be scheduled for publication until you have made the required changes, so a swift response is appreciated.

IMPORTANT: The editorial review process is now complete. PLOS will only permit corrections to spelling, formatting or significant scientific errors from this point onwards. Requests for major changes, or any which affect the scientific understanding of your work, will cause delays to the publication date of your manuscript.

Should you, your institution's press office or the journal office choose to press release your paper, you will automatically be opted out of early publication. We ask that you notify us now if you or your institution is planning to press release the article. All press must be co-ordinated with PLOS.

Thank you again for supporting Open Access publishing; we are looking forward to publishing your work in PLOS Computational Biology.

Best regards,

Philipp Martin Altrock, Ph.D.

Academic Editor

PLOS Computational Biology

Tobias Bollenbach

Section Editor

PLOS Computational Biology

***********************************************************

# Editor report: Acceptance letter

**DOI:** 10.1371/journal.pcbi.1014258.r008

**Authors:** Philipp Altrock, Tobias Bollenbach

PCOMPBIOL-D-26-00932R3

Dynamic Workforce Modulation and Foraging Efficiency in Eusocial Insect Colonies

Dear Dr Campos,

I am pleased to inform you that your manuscript has been formally accepted for publication in PLOS Computational Biology. Your manuscript is now with our production department and you will be notified of the publication date in due course.

The corresponding author will soon be receiving a typeset proof for review, to ensure errors have not been introduced during production. Please review the PDF proof of your manuscript carefully, as this is the last chance to correct any errors. Please note that major changes, or those which affect the scientific understanding of the work, will likely cause delays to the publication date of your manuscript.

Soon after your final files are uploaded, unless you have opted out, the early version of your manuscript will be published online. The date of the early version will be your article's publication date. The final article will be published to the same URL, and all versions of the paper will be accessible to readers.

For Research, Software, and Methods articles, you will receive an invoice from PLOS for your publication fee after your manuscript has reached the completed accept phase. If you receive an email requesting payment before acceptance or for any other service, this may be a phishing scheme. Learn how to identify phishing emails and protect your accounts at https://explore.plos.org/phishing.

Thank you again for supporting PLOS Computational Biology and open-access publishing. We are looking forward to publishing your work!

With kind regards,

Sharmila Kamatchi

PLOS Computational Biology | Carlyle House, Carlyle Road, Cambridge CB4 3DN | United Kingdom ploscompbiol@plos.org | Phone +44 (0) 1223-442824 | ploscompbiol.org | @PLOSCompBiol
